# Exacerbated age-related hearing loss in mice lacking the p43 mitochondrial T3 receptor

**DOI:** 10.1186/s12915-021-00953-1

**Published:** 2021-02-01

**Authors:** Corentin Affortit, François Casas, Sabine Ladrech, Jean-Charles Ceccato, Jérôme Bourien, Carolanne Coyat, Jean-Luc Puel, Marc Lenoir, Jing Wang

**Affiliations:** 1 INSERM - UMR 1051, Institut des Neurosciences de Montpellier, 80 rue Augustin Fliche, 34295 Montpellier, France; 2 Université de Montpellier, 34000 Montpellier, France; 3 INRA, UMR 866 Dynamique Musculaire et Métabolisme,, 34060 Montpellier, France; 4 ENT Department, CHU Montpellier, 34295 Montpellier, France

**Keywords:** Age-related hearing loss, Thyroid hormones, p43 mitochondrial T3 receptor, Mitochondrial dysfunction

## Abstract

**Background:**

Age-related hearing loss (ARHL), also known as presbycusis, is the most common sensory impairment seen in elderly people. However, the cochlear aging process does not affect people uniformly, suggesting that both genetic and environmental (e.g., noise, ototoxic drugs) factors and their interaction may influence the onset and severity of ARHL. Considering the potential links between thyroid hormone, mitochondrial activity, and hearing, here, we probed the role of p43, a N-terminally truncated and ligand-binding form of the nuclear receptor TRα1, in hearing function and in the maintenance of hearing during aging in p43^−/−^ mice through complementary approaches, including in vivo electrophysiological recording, ultrastructural assessments, biochemistry, and molecular biology.

**Results:**

We found that the p43^−/−^ mice exhibit no obvious hearing loss in juvenile stages, but that these mice developed a premature, and more severe, ARHL resulting from the loss of cochlear sensory outer and inner hair cells and degeneration of spiral ganglion neurons. Exacerbated ARHL in p43^−/−^ mice was associated with the early occurrence of a drastic fall of SIRT1 expression, together with an imbalance between pro-apoptotic Bax, p53 expression, and anti-apoptotic Bcl2 expression, as well as an increase in mitochondrial dysfunction, oxidative stress, and inflammatory process. Finally, p43^−/−^ mice were also more vulnerable to noise-induced hearing loss.

**Conclusions:**

These results demonstrate for the first time a requirement for p43 in the maintenance of hearing during aging and highlight the need to probe the potential link between human *THRA* gene polymorphisms and/or mutations and accelerated age-related deafness or some adult-onset syndromic deafness.

**Supplementary Information:**

The online version contains supplementary material available at 10.1186/s12915-021-00953-1.

## Background

Age-related hearing loss (ARHL), or presbycusis, is the hearing loss that occurs gradually in most people, as they age. This type of hearing loss is generally associated with difficulty in speech discrimination, as well as in sound detection and localization, particularly in noise. Unmanaged presbycusis may contribute to social isolation, cognitive decline, and dementia [1]. However, the age of onset and severity of ARHL is also highly variable. This is probably due to the complexity of intrinsic (genetic predisposition) and external (e.g., noise, ototoxic drugs) factors and their interaction. To date, the exact mechanisms driving the age-related degeneration of the cochlear structures remain poorly understood.

Thyroid hormones play essential roles in the regulation of many processes in the development of mammals at stages before the onset of function in a variety of organ systems, including the inner ear [2]. Thyroid hormones are also key regulators of mitochondrial activity [3]. Triiodothyronine (T3) is considered to be the main, active hormone. The actions of T3 are mediated by two nuclear thyroid hormone receptors (TRβ and TRα), encoded by the *Thrb* and *Thra* genes, respectively [4]. Both receptors are mainly expressed in the cochlea during development before the onset of hearing function [2, 5, 6]. *Thrb* differentially expresses two N-terminal isoforms, TRβ1 and TRβ2, and *Thra* expresses TRα1 and TRα2, a splice variant, in overlapping patterns in several cochlear locations [7, 8]. The deficiency of TRβ induces deafness and thyroid hyperactivity in humans [9] and mice [10–12]. In contrast, TRα has not been considered to be critical for hearing function [13]. However, this idea has been challenged by data revealing that mice carrying a TRα1 point mutation exhibit auditory defects and a range of middle-ear abnormalities [8]. An even worse cochlear phenotype has also been described in TRα/TRβ double-knockout mice [13]. In addition, a role of TRα1 in auditory function was suggested in a study showing that in mice, the *Thra*^tm2^ mutation leads to the deletion of TRα2, together with increased expression of TRα1. Furthermore, the introduction of a *Thra*^tm2^ allele in *Thrb*-null mice rescued hearing and thyroid phenotypes caused by the absence of TRβ [14]. In addition, previous studies also demonstrated a role for TRα1 in the final differentiation of outer hair cells (OHCs) [15] and the requirement of the thyroid hormone for the normal molecular, morphological, and functional maturation of IHC ribbon synapses [16, 17].

p43 is a mitochondrial T3 receptor encoded by TRα1 mRNA that is imported into mitochondria and located in their matrices [18]. P43 is a N-terminally truncated TRα1 that is synthesized by the use of alternative initiation site of translation in the TRα1 transcript [19]. In the presence of T3, p43 stimulates mitochondrial activity and mitochondriogenesis [19, 20]. P43 acts as a mitochondrial transcription factor, inducing changes in the mitochondrial/nuclear crosstalk by increasing mitochondrial activity, thus significantly increasing the number of genes targeted by T3, relative to the number of genes directly targeted by T3 nuclear receptors [18]. Mice with a selective deletion of p43 showed decreased mitochondrial respiratory chain activity, a major defect in insulin secretion leading to glucose intolerance, and insulin resistance during aging [21]. These mice also had slightly greater amounts of thyroid hormones [21].

Considering the potential links between thyroid hormone, mitochondrial activity, and hearing, here we probe the role of p43 in hearing function and in the maintenance of hearing during aging in p43^−/−^ mice in a C57bl/6 J background. These mice carry a specific p43 invalidation but that still expressed ΤRα1 and TRα2. In addition, ΤRα1 transcriptional activity was not affected by p43 deletion [22]. Based on complementary approaches combining morpho-physiology, biochemistry, and molecular biology, we show that in juvenile mice, the selective deletion of p43 caused no obvious hearing loss. However, over subsequent months, these mice developed a premature and more severe ARHL. The premature hearing loss is mainly due to the accelerated loss of outer hair cells (OHCs), inner hair cells (IHCs), and spiral ganglion neurons (SGNs). Finally, p43^−/−^ mice were also more sensitive to noise damage. Together, these results suggest an important role for p43 in the maintenance of hearing against the effects of aging and lifetime noise exposure in adulthood.

## Results

### P43 deletion exacerbates age-related hearing impairments

The generation of p43 knockout mice (p43^−/−^) that still expressed ΤRα1 and TRα2 was demonstrated by Blanchet et al., [22]. Here, we found that TRα 1,2 immunoreactivity was present in both the cytoplasm and nuclei of IHCs, OHCs, and SGNs, but only in the nuclei of their supporting cells in WT mice (Additional file 1: Fig. S1A-F). P43^−/−^ mice displayed a reduction in TRα immunoreactivity in the cytoplasm of OHCs and IHCs and, to a lesser extent, in those of SGNs, but not in the nuclei of the sensory neural cells and supporting cells (Additional file 1: Fig. S1G-L), thus suggesting that p43 deletion did not affect nuclear TRα expression.

**Fig. 1:**
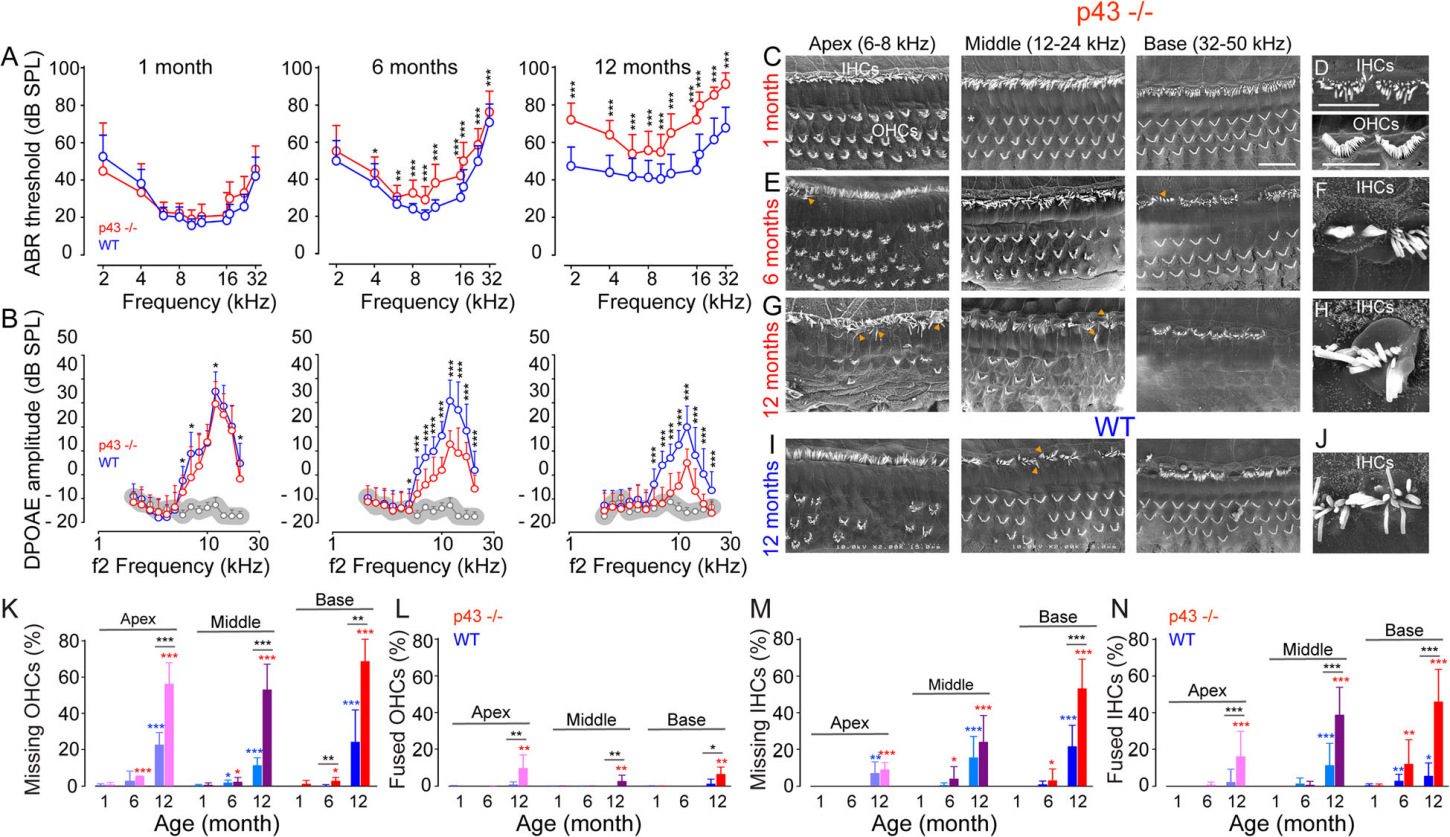
Exacerbated age-related hearing and hair cell loss in p43^−/−^ mice. **a** Age-related auditory brainstem response (ABR) thresholds. Note the high- to low-frequency gradient of the deterioration of ABR thresholds in both strains, but more prominent in p43^−/−^ (red plot) than in WT (blue plot) mice. **b** Age-related distortion product otoacoustic emission (DPOAE) amplitudes. Note that the DPOAE amplitudes decrease with age in both strains, but more severe in p43^−/−^ (red plot) than in WT (blue plot) mice. The gray dots and shaded area represent the background noise levels. Data are expressed as mean ± SD (1 month: *n* = 31; 6 months: *n* = 23; 12 months: *n* = 15 mice per strain). One-way ANOVA test followed by *Dunn’s* test (*P ≤ 0.05, **P ≤ 0.01, ***P ≤ 0.001, p43^−/−^ vs. WT mice). **c e**, **g**, **i** Representative scanning electron microscopy (SEM) micrographs showing the apical, mid, and basal cochlear regions from p43^−/−^ (**e**, **e**, and **g**) and WT (**i**) at 1 (**c**), 6 (**e**), and 12 (**g** and **i**) months of age. **d**, **f**, **h, j** Higher magnification of SEM images of representative OHC and IHC stereocilia from p43^−/−^ (**d**, **f,** and **h**) and WT (**j**) at 1 (**d**), 6 (**f**), and 12 (**h** and **j**) months of age. Note the fused stereocilia of IHCs in **f**, **h**, and **j**. Scale bars: **c**, **e**, **g**, and **i** = 25 μm; **d**, **f**, **h**, and **j** = 10 μm. OHCs, outer hair cells; IHCs, inner hair cells; white star indicating OHC loss; yellow arrows pinpointing stereocilial fusion. **k**–**n** Cytocochleograms representing the percentage of missing OHCs (**k**) and IHCs (**m**), and fused OHCs (**l**) and IHCs (**n**) in apical, mid, and basal regions from the cochleae of p43^−/−^ (red bars) and WT (blue bars) aged 1, 6, and 12 months. Data are expressed as mean ± SD (*n* = 6 to 12 mice per age and strain). One-way ANOVA test was followed by *Dunn’s* test. *P ≤ 0.05, **P ≤ 0.01, ***P ≤ 0.001, p43^−/−^ vs. WT mice of the same age. *P ≤ 0.05, **P ≤ 0.01, ***P ≤ 0.001, older p43^−/−^ vs. 1-month-old p43^−/−^. *P ≤ 0.05, **P ≤ 0.01, ***P ≤ 0.001, older WT vs. 1-month-old WT

To assess the effect of p43 deletion on hearing and on the maintenance of hearing during aging, we recorded the auditory brainstem responses (ABRs), which reflect the synchronous activation of auditory neurons from the cochlea up to the colliculi in response to incoming sound, and the distortion product otoacoustic emissions (DPOAEs) reflecting the normal function of OHCs, in both WT and p43^−/−^ mice during aging. Our results showed that ABR thresholds in p43^−/−^ mice were virtually identical to WT at 1 month of age (Fig. 1a). The amplitude of the ABR wave I can provide an objective measure of the loss of IHC ribbon-synapse function when measured at a high sound level greater than 70 dB SPL. Here, we show that at 1 month of age, the ABR wave-I amplitudes were similar at all sound pressures tested between the strains (Additional file 1: Fig. S2C).

At later ages, both strains showed the progressive, typical age-related increase of ABR thresholds, beginning at high frequencies and progressing towards low frequencies. Significantly, from 6 months of age, significantly higher ABR thresholds at frequencies from 4 to 32 kHz were observed in p43^−/−^ mice when compared to WT animals (*P* ≤ 0.05, Fig. 1a). At this stage, an age-related decrease in ABR wave-I amplitudes was seen in both strains, but more severely in p43^−/−^ than in WT mice (*P* ≤ 0.01, Additional file 1: Fig. S2D). At 12 months, significantly higher ABR thresholds were observed at all frequencies tested in p43^−/−^ compared to WT mice (*P* ≤ 0.001, Fig. 1a). At 18 months of age, the mutant mice had already completely lost their hearing, while the WT mice were profoundly deaf (Additional file 1: Fig. S2E).

At 1 month of age, a slight, but significantly reduced DPOAE amplitude was observed at the frequencies 6, 7, 12, and 20 kHz in p43^−/−^ compared with WT mice (*P* ≤ 0.05, Fig. 1b). At later stages, both strains developed progressive, age-related loss of DPOAE amplitudes. Significant reductions of the DPOAE amplitudes were observed at frequencies ranging from 5 to 20 kHz in p43^−/−^ mice compared to WT mice from 6 months of age (*P* ≤ 0.05) and maintained to 12 months (Fig. 1b).

The endocochlear potential (EP), reflecting the functional state of the stria vascularis, was maintained in both WT and p43 KO mice until 12 months of age (Additional file 1: Fig. S2F); together with no evident ultrastructural abnormality observed in the stria vascularis and fibrocytes in both strains over the same time span (Additional file 1: S2G), we propose that p43 deletion exacerbates ARHL through mechanisms driving the death of sensory hair cells and SGNs.

### Hair-cell loss with age in p43^−/−^ mice

#### Scanning electron microscopy (SEM) assessments

Eleven days after birth, p43^−/−^ mice showed a normal appearance of the surface morphology and the overall organization of the organ of Corti under SEM observation (Additional file 1: Fig. S2A-B). The stereociliary bundles of hair cells displayed the characteristic shapes—straight for IHC and V-shaped for OHC, and were of uniform orientation (Additional file 1: Fig. S2A-B). At 1 month of age, a sporadic loss of OHCs was occasionally observed (Fig. 1c, d). At 6 months of age, degeneration of OHCs was observed throughout the whole cochlea, in addition to fusion of stereocilia of IHC, that mainly occurred in the basal region of the cochleae (Fig. 1e, f). At 12 months of age, the majority of OHC hair bundles were missing, and those that remained showed extensive stereocilial fusion (Fig. 1g). There were large amounts of IHC loss, mainly in basal region, and in addition, the remaining IHCs often had fused stereocilia (Fig. 1g, h). At this age, an age-related loss of the OHCs and some IHCs, and fusion of stereocilia of the remaining IHC, was also observed in WT mice, although to a lesser extent (Fig. 1i, j).

Counts of the sensory hair cells showed that both strains developed age-related OHC loss, but more severely in p43^−/−^ mice (mean ± SD = 68.5% ± 12.4 versus 24.1% ± 17.8 OHC loss in the basal region in p43^−/−^ and WT mice at 12 months, respectively, *P* ≤ 0.01, Fig. 1k). At 12 months of age, the cochleae of p43^−/−^ mice showed a significant increase in numbers of remaining OHCs that had fused stereocilia (*P* ≤ 0.05 or 0.01 vs. 1 month, or vs. WT mice of the same age, Fig. 1l).

A significant increase (*P* ≤ 0.01) in IHC loss occurred at 12 months of age in both strains, but was more severe in the basal region of p43^−/−^ compared to WT mice (mean ± SD = 53% ± 16.1 versus 21.4% ± 11.6 for p43^−/−^ and WT mice, respectively, *P* ≤ 0.001, Fig. 1m). A significant increase in numbers of the IHCs with fused stereocilia occurred early (6 months) in the cochlear basal region of both strains (*P* ≤ 0.01 vs. 1 month, Fig. 1n). At 12 months of age, significantly higher numbers (*P* ≤ 0.001) of IHCs with fusion of their stereocilia were observed in whole cochlea of p43^−/−^ (basal region: mean ± SD = 45.8 ± 17.8) compared to WT mice (basal region: mean ± SD = 5.4% ± 7.2).

Altogether, these results indicate that the deletion of p43 enhanced the age-related loss of OHCs and, to a lesser extent, of IHCs in mice.

#### Transmission electron microscopy (TEM) evaluation

**Fig. 2:**
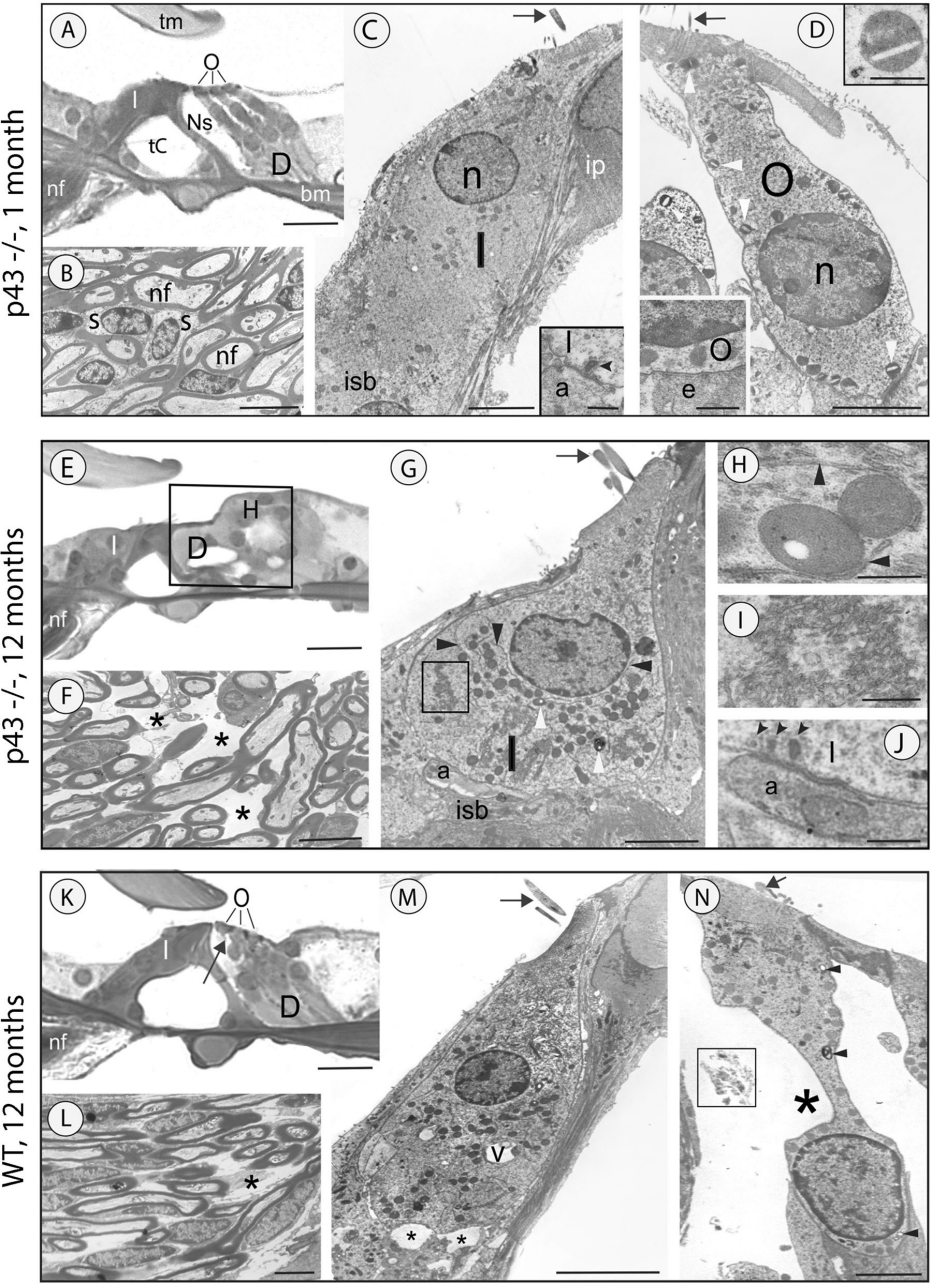
Ultrastructural changes in the organ of Corti. Representative light (**A**, **B**, **E**, **F**, **K**, **L**) and transmission electron (**C**, **D**, **G**–**J**, **M**, **N**) micrographs show the organ of Corti from p43^−/−^ (**A**–**J**) and WT mice (**K**–**N**). **A**–**D** 1-month-old p43^−/−^ mice. The organ of Corti shows adult-like structural characteristics with well-opened tunnel of Corti (tC) and spaces of Nuel (Ns) and normally shaped tectorial (tm) and basilar (bm) membranes (**A**). The 3 OHCs (O) above the Deiters cells (D), and the IHC (I) are present (**A**). Densely packed myelinated nerve fibers (nf) and schwann cells (s) are visible in the spiral lamina (**B**). The nucleus (n) of IHC is well positioned in the upper half of the cell body (**C**). The stereocilia (arrow) are erect. Beneath the basal pole of the IHC, the inner spiral bundle (isb) contains numerous nerve fibers. The inset in **C** shows a typical synaptic contact between the IHC and the extremity of a radial afferent fiber (a). Note the synaptic ribbon (arrowhead) within the IHC. ip, inner pillar cell. Note the apparently normal OHC with erect stereocilia (arrow) and basally located nucleus (**D**). Most of its mitochondria are damaged (white arrowheads and upper inset in **D**). Lower inset in **D**: a typical efferent synapse at the basal pole of a neighboring OHC (e: efferent ending). **E**–**J** 12 months of age p43^−/−^ mice. Shown is an organ of Corti containing IHC, but lacking OHC (**E**). In the area of the missing OHCs (delineated by the black square), an epithelial scar is formed by Deiters (D) and Hensen (H) cells (**E**). In the osseous spiral lamina, empty spaces (asterisks) are visible in the bundle of nerve fibers (**F**). The IHC is deformed and contains damaged mitochondria (white arrowheads), an exuberant network of endoplasmic reticulum (black arrowheads) contacting the mitochondria, and large aggregates of membranous material resembling reticulum debris (within the black square, **G**). The inner spiral sulcus contains only few afferent (a) nerve fibers (**G**). **H**–**J** Enlargement of organelles seen in the IHC shown in **G**. **H** Two closely linked mitochondria with the absence of their cristae, and a portion of the lower mitochondrion is free of matrix. Note well-defined endoplasmic reticulum (arrowhead) in contact with the two mitochondria. **I** Accumulation of membranous material such as endoplasmic reticulum debris. **J** Immature-like afferent synapse at the basal pole of the IHC. Note the multiple synaptic ribbons (arrowheads) in the IHC and the elongated postsynaptic density in the afferent fiber. **K–N**: 12 months of age WT mice. **K** The organ of Corti contains the IHC and the OHCs, but the OHC from the first row is damaged (arrow). **L** Among the nerve fibers, some empty spaces (asterisk) are visible in the spiral lamina. **M** The IHC has a typical shape and erect stereocilia (arrow). Note the large autophagic vacuoles (v) within the cytoplasm of the cell and the swollen afferent dendrite extremities (asterisks) at its basal pole. **N** OHC from the first row, showing a distorted cell body (asterisk), bent and fused stereocilia (arrow) and some damaged mitochondria (arrowheads). Note the cell debris (in the black square) floating within the spaces of Nuel. **A**, **E**, **K** = 50 μm. **C**, **G**, **M** = 10 μm. **B**, **D**, **F**, **L**, *N* = 5 μm. **H**, **J** = 1 μm. **I** = 0,5 μmm. Inset **C** and lower inset **D = 1** μm. Upper inset **D** = 0.5 μm. *N* = 4 cochleae per age and strain

In radial sections of the organ of Corti from 1-month-old P43^−/−^ mice, one IHC and three elongated OHCs were separated by a widely opened tunnel of Corti, and the OHCs were individualized into a well-formed space of Nuel (Fig. 2A). At the entrance to the organ of Corti, the spiral lamina contained a normal density of myelinated nerve fibers (Fig. 2B).

TEM investigations confirmed that both types of hair cells looked normal (Fig. 2C, D). In the IHC, mitochondria also looked normal. In contrast, the OHCs contained numerous damaged mitochondria (Fig. 2D and upper insert). Both types of hair cells showed well-formed synaptic contacts at their basal pole (lower inserts in Fig. 2C, D). The IHCs were contacted by several dendrites from type I spiral ganglion neurons; each afferent synapse typically showed one well-defined presynaptic ribbon and a postsynaptic membrane thickening (insert Fig. 2C). The OHCs essentially showed one or two large synapses with the axons from the medial efferent system (lower insert Fig. 2D). These efferent synapses showed the classical presynaptic vesicle aggregates and an extended postsynaptic cistern. The ultrastructural abnormalities of mitochondria included size enlargement, disappearance of the cristae, and presence of large vacuoles in the matrix (upper insert Fig. 2D).

In 12-month-old p43^−/−^ mice, the cytoarchitectural organization of the organ of Corti was profoundly affected in the OHC region, due to massive loss of OHCs and subsequent formation of an epithelial scar (Fig. 2E). The region of the IHC was unchanged, but some nerve fibers were missing in the spiral lamina (Fig. 2F). TEM evaluation showed that the remaining IHCs displayed a deformed cell body (Fig. 2G), alteration of mitochondrial cristae and matrix (Fig. 2G-H), increase in the size of mitochondria (Figs. 2H and 5a), and cytoplasmic accumulation of membranous debris (Fig. 2G, I). At the basal pole of the IHC, we observed immature-like afferent synapses (Fig. 2G, J) that were characterized by the presence of multiple pre-synaptic bodies, and afferent dendrites with an elongated profile resembling that of growing fibers. These features suggested a process of neo-synaptogenesis as it happens after excitotoxicity when injured afferent synapses are replaced by new ones [23].

In 12-month-old WT mice, the organ of Corti had a normal aspect, although some OHCs looked damaged (Fig. 2K). In the spiral lamina, a few nerve fibers were missing (Fig. 2L). TEM assessments showed that the IHCs had a normal shape, but vacuoles of various sizes were present in the cytoplasm (Fig. 2M). Beneath the basal pole of the IHCs, occasional swollen afferent dendrites were present (Fig. 2M), suggesting that some type I spiral ganglion neurons had suffered an aging-related susceptibility to excitotoxicity as demonstrated in aged hippocampal neurons in culture [24]. Some OHCs showed a distorted cell body and flattened stereocilia and their cytoplasm contained a number of abnormal mitochondria (Fig. 2N).

### Loss and ultrastructural changes of spiral ganglion neurons with age in p43^−/−^ mice

**Fig. 3:**
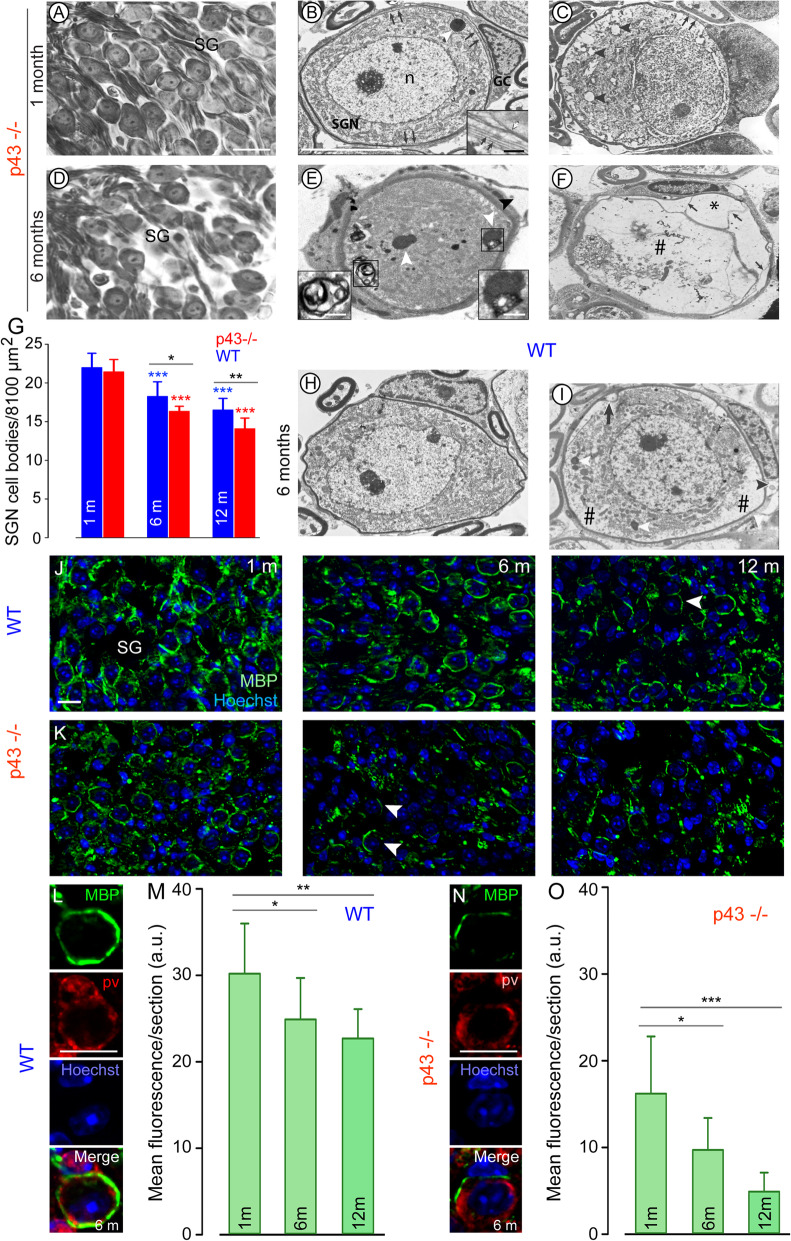
Degeneration of the spiral ganglion neurons and their glial cells. Representative light (**a**, **d**), transmission electron (**b, c**, **e**, **f**, **h**, **i**) and confocal (**j**, **k**, **l**, **n**) micrographs showing the spiral ganglion neurons and their glial cells from p43^−/−^ (**a**–**f**, **k**, **n**) and WT mice (**h**, **i**, **j**, **l**), quantitative analysis of SGN density (**g**) and semi-quantitative analysis of and MBP expression (**m**, **o**). **a**–**c** 1 month of age p43^−/−^ mice. **a** Micrograph showing a normal ganglion cell density. **b** The spiral ganglion neuron (SGN) have a healthy appearance. In the glial cells (GC), the myelin sheath is disorganized (arrows). Inset in **b** Disorganized myelin sheath (white arrow) with several layers of non-compacted myelin (black arrows). n, nucleus of the SGN. **c** The cytoplasm of the SGN shows numerous autophagic vesicles (arrowheads) and a ring of perinuclear edema. Arrows indicate disrupted myelin. **d**–**f** 6 months of age p43^−/−^ mice. **d** A decrease in ganglion cell density is shown. **e** The SGN has condensed cytoplasm, a number of dark inclusions (white arrowheads) resembling lipofuscin aggregates and also a typical autophagy double-membrane vesicle (delineated by the black square). Note the detached myelin sheath from its cell body. Black arrowhead indicates a glial cell with large vacuoles in the cytoplasm. Right Inset shows a dark inclusion with a lipid droplet (delineated by the black square in **e**). Left Inset: shows an autophagy double-membrane vesicle. **f** The SGN cytoplasm shows large areas of autolysis (#). The glial cell still surrounds the remnants of the SGN but the myelin envelope is cleaved leading to large empty spaces between the myelin sheets (asterisk). Note the strands of isolated myelin sheets (arrows) and the autophagic vacuole in the glial cell. **h**, **i** 6 months of age WT mice. **h** A healthy appearing SGN surrounded by a normal looking glial cell with well compacted myelin sheath. **I** The cytoplasm of the SGN presents areas of autolysis (#) and dark inclusions (white arrowheads). The glial cell shows an autophagic vacuole (arrowhead). The myelin sheath is cleaved (arrow) in several segments. Scale bars: **a**, **d** = 20 μm, **b**, **c**, **e**, **f**, **h**, **i** = 10 μm. Insets in **b**, **e** = 0.5 μm. **g** Histogram representing the average SGN density in Rosenthal’s canal of the basal region from p43^−/−^ (red bars) and WT (blue bars) aged 1, 6, and 12 months. Data are expressed as mean ± SD (*n* = five sections per cochlea, 7 to 9 cochleae per age and strain). One-way ANOVA test was followed by *Dunn’s* test (*P = 0.03, **P ≤ 0.01, p43^−/−^ vs. WT of the same age; ***P ≤ 0.001, older p43^−/−^ vs. 1-month-old p43^−/−^; ***P ≤ 0.001, older WT vs. 1-month-old WT). **j**, **k**, **l**, **n** Confocal images showing the basal region of spiral ganglion (SG) immunolabeled for parvalbumin (PV) to identify SGNs (red, not shown in **j** and **k**), and MBP (green) to highlight myelin sheaths, and counterstained with Hoechst (blue) to identify nuclei from WT (**j**, **l**), and p43^−/−^ (**k**, **n**) aged 1, 6, and 12 months (left, middle and right columns respectively). Higher magnification images in **l** and **n** show representative intact (**l**) and partial loss of (**n**) MBP positive myelin sheaths enveloping the neurons from WT (**l**) and p43^−/−^ (**n**) mice aged 6 months. Scale bars = 20 μm. **m**, **o**: Semi-quantitative analysis of the MBP immunofluorescence. Mean immunofluorescence of myelin per section as a function of age, for WT (**m**) and p43^−/−^ (**o**). Data are expressed as mean ± SD (*n* = 3 sections per cochlea, 4 cochleae per age and strain). Wilcoxon test (*P ≤ 0.05, **P ≤ 0.01, ***P ≤ 0.001)

One-month-old p43^−/−^ mice showed a healthy morphological appearance and density of SGNs (Fig. 3a). At 6 months, the SGN density was reduced, mainly in the basal region (Fig. 3d). The measurement of SGN density revealed that at 1 month of age, a slight, but not significant, reduction in density of SGNs was observed in p43^−/−^ mice compared to in WT (Fig. 3g). At later stages, both strains developed a progressive, age-related loss in SGN density, but more severely in p43^−/−^ than in WT animals (6 months: *P* ≤ 0.05, 12 months: *P* ≤ 0.01, Fig. 3g).

At 1 month of age, the general morphology of the spiral ganglion neurons and their glial cells was well preserved in p43^−/−^ mice. The glial cell-derived myelin envelope, which surrounds the neurons, was well developed (Fig. 3b). However, at the ultrastructural level, discrete changes were evident in both types of cells. Prominently, in all glial cells, short segments of myelin showed non-compacted and split lamellae (insert Fig. 3b). In numerous, but not all, neurons, autophagic vacuoles were abundant (Fig. 3c).

At 6 months of age, p43^−/−^ mice displayed several forms of severe alterations in all SGNs and in their glial cells (Fig. 3e, f). The accumulation of aggregates of electron-dense material, integrating lipid droplets (Fig. 3e and right insert), typical double-membrane autophagosomes (Fig. 3e and left insert), and the moderate-to-large areas of autolytic damage (Fig. 3f) was often observed in the cytoplasm of the SGNs. In the glial cells, we noted an aggravation of the splitting process that could produce large bulges in the myelin sheaths (Fig. 3e, f) and the frequent presence of large, autophagic vacuoles in the cytoplasm of the glial cells (Fig. 3e, f).

In the cochleae of 1-month-old WT mice (not shown), all SGNs and glial cells had a healthy appearance. In 6-month-old control animals, beside healthy looking SGNs and glial cells (Fig. 3h-i), we observed SGNs showing small areas of autolysis, and glial cells containing large autophagic vacuoles (Fig. 3i).

### Declines of myelin basic protein immunoreactivity in p43^−/−^ mice

To confirm myelin abnormalities, we investigate myelin basic protein (MBP), which is the main component of the myelin sheath in the peripheral nervous system [25]. Our results revealed intense immunostaining for MBP surrounding SGNs and their processes in the SGNs of WT mice aged 1 and 6 months (Fig. 3j). In these young WT cochleae, the MBP myelin sheath was intact and enclosed the entire SGN (Fig. 3j, l). However, by 12 months, the MBP myelin sheaths in many neurons were discontinuous and, in some cases, missing completely in WT mice (Fig. 3j). By contrast, abnormalities in the MBP staining pattern were observed early in the SGNs of p43^−/−^ mice, even at 1 month of age, and worsening with age (Fig. 3k–n).

Semi-quantitative analysis of the immunofluorescence per section in the basal cochlear regions showed a significant age-related decrease in MBP abundances in both WT and p43^−/−^ mice (*P* ≤ 0.05 vs. 1 month, Fig. 3m, o). In addition, SGNs from p43^−/−^ mice always had lower MBP immunofluorescence for all ages tested (1 month: mean ± SD = 30.2 ± 5.8 a.u. vs. 16.2 ± 6.6 a.u for WT and p43^−/−^, respectively, *P* = 0.0019; 6 months: 24.9 ± 4.8 a.u. vs. 9.7 ± 3.7 a.u for WT and p43^−/−^, respectively, *P* = 0.0003; 12 months: 22.7 ± 3.4 a.u. vs. 4.9 ± 2.2 a.u for WT and p43^−/−^, respectively, *P* = 0.0002, Fig. 3m, o).

These results, supported by TEM examination, established the earlier occurrence of pathological changes in the myelin sheaths of p43^−/−^ mice.

### Functional and morphological correlate

**Fig. 4:**
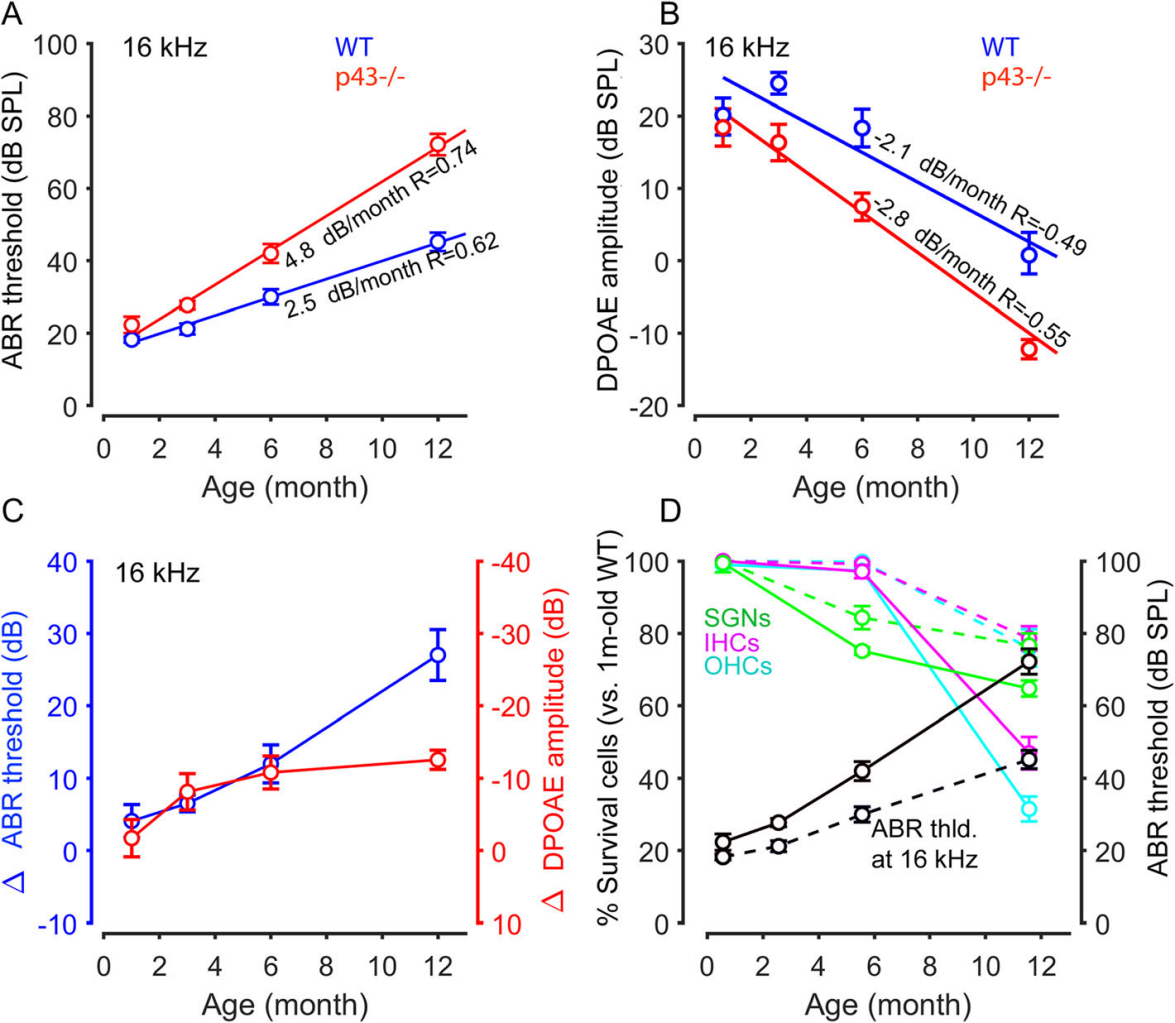
Age-related hearing impairments and morphological correlation. **a**, **b** Age-related elevation of mean ABR thresholds (**a**) and reduction of DPOAE amplitudes (**b**). Red and blue circles represent the values of mean ABR threshold at 16 kHz (in **a**) and the mean DPOAE amplitude at 16 kHz (in **b**) for each mouse and each time point. These values were then used to perform a linear regression, allowing the measurement of threshold elevation or amplitude reduction per month and the calculation of Pearson correlation coefficients. **c** Time course of ABR threshold increase and DPOAE amplitude decrease in the p43^−/−^ mice. The left ordinate represents the mean difference (Δ) in ABR thresholds between p43^−/−^ and WT mice. The right ordinate represents the mean difference (Δ) in DPOAE amplitudes between p43^−/−^ and WT mice. The abscissa represents the time in months. The data were derived from **a** and **b**. **d** Elevation of ABR thresholds (black circles) at 16 kHz relating to the percentage of OHC (cyan circles), IHC (magenta circles), and SGN survival (green circles) in the cochlear basal region from p43^−/−^ (solid line) and WT (dotted line) mice aged 1, 6, and 12 months. The left Y-axis represents the mean percentage of remaining OHCs, IHCs and SGNs related to 1-month-old WT mice. The right Y-axis represents the values of mean ABR threshold

The ABR threshold showed no significant difference between p43^−/−^ and WT mice at 1 month of age (Fig. 4a). In contrast, both strains of mice showed an age-related hearing loss that was more severe in p43^−/−^ mice (4.8 dB/month, *R* = 0.74 at 16 kHz, *P* ≤ 0.001) than in WT mice (2.5 dB/month, *R* = 0.62 at 16 kHz, *P* ≤ 0.001, Fig. 4a). In addition, an age-related decrease in DPOAE amplitude occurred faster in p43^−/−^ mice (− 2.8 dB/month, *R* = 0.55 at 16 kHz, *P* ≤ 0.001) than in WT mice (− 2.1 dB/month, *R* = 0.49 at 16 kHz, *P* ≤ 0.001, Fig. 4b). To facilitate the comparison between the two strains, we then expressed the loss of DPOAE amplitudes and the increase of ABR thresholds with respect to the control WT as a function of age. The behavior of the ABR thresholds and DPOAE amplitudes in p43^−/−^ mice were similar up to 6 months of age (Fig. 4c). At 12 months of age, however, a greater effect on ABR thresholds was observed. A possible explanation is that additional neural damage occurred in the 12-month-old KO mice, more severely affecting ABR responses than DPOAEs, since the latter only reflect OHC damage.

We then expressed in percent survival of OHCs, IHCs, and SGNs during aging by normalizing them to those of 1-month-old WT mice, and an increase in ABR thresholds linked to age in WT and p43^−/−^ mice. While both strains displayed comparable amounts of OHC, IHC, and SGN survival and ABR thresholds at 1 month (Fig. 4d), by 6 months, they displayed an age-related decrease in survival of SGNs, to a lesser degree of IHCs and OHCs, and an increase in the ABR thresholds that were more important in p43−/− than in WT mice. At 12 months old, the progression of SGN loss continued slowly in both strains (more noticeably in p43^−/−^ than WT mice). However, in the in p43^−/−^ mice, we observed a dramatic decrease in the survival of OHCs, and to a lesser extent of IHCs, as well as a dramatic increase in the ABR threshold (Fig. 4d).

Collectively, these data indicate that a greater loss of OHCs and IHCs, and, to a lesser extent SGNs, contributed to the enhanced ARHL in p43^−/−^ mice.

### Mitochondrial dysfunction, oxidative stress, and decreased expression of Sirtuin 1 in p43^−/−^ mice

**Fig. 5:**
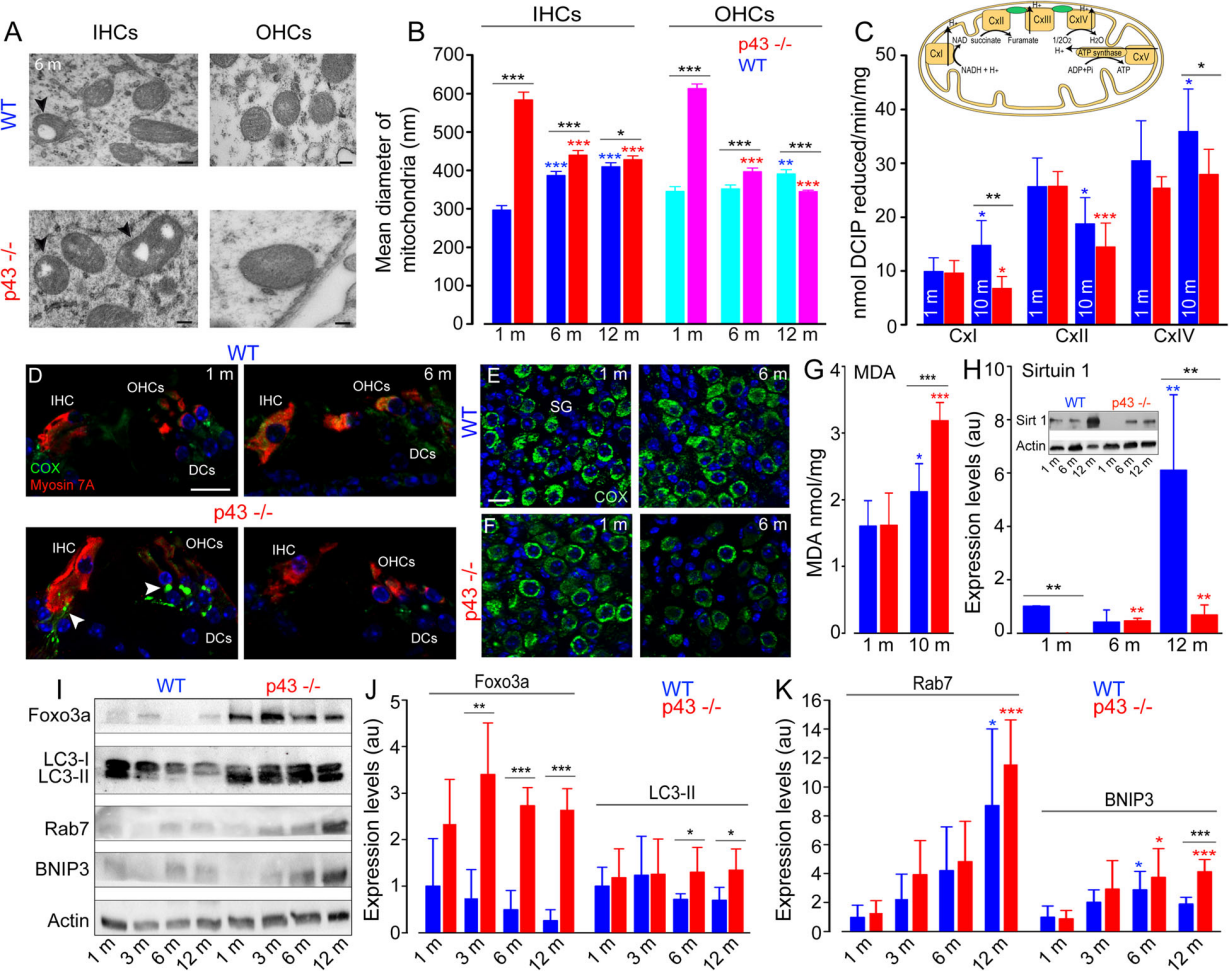
Increase of mitochondrial size and dysfunction, oxidative stress and impaired SIRT1 expression and autophagic activities. **a** Representative transmission electron (TEM) micrographs showing the mitochondria located in the IHCs and OHCs from WT and p43^−/−^ mice aged 6 months. Note the increase in the number of mitochondria with the lack of their cristae (arrowheads) and enlarged mitochondria in the hair cells of p43^−/−^ mice. Scale bars = 0.5 μm. **b** The histogram represents the mean diameter of the mitochondria in the IHCs and OHCs from p43^−/−^ (red bars) and WT (blue bars) mice obtained by TEM imaging measurements. Data are expressed as mean ± SD (n = ~ 45 to 50 mitochondria, taken randomly from the IHCs or OHCs, 4 cochleae per age and strain). One-way ANOVA was followed by *Dunn’s* test (*P = 0.014, ***P ≤ 0.001, p43^−/−^ vs. WT of the same age; ***P ≤ 0.001, older p43^−/−^ vs. 1-month-old p43^−/−^; **P ≤ 0.01, ***P ≤ 0.001, older WT vs. 1-month-old WT). Inset in **c** Schematic representation of the mitochondrial respiratory chain. Complex I (CxI, NADH dehydrogenase) is the entry point of electron transfer in the respiratory chain. The proton (H+) gradient generated at the level of complex I, III (CxIII, cytochrome-c reductase) and IV (CxIV, cytochrome-c oxidase) is used by ATP synthase for ATP synthesis. Complex II (CxII, succinate dehydrogenase) is a central driver of the reprogramming of metabolic and respiratory adaptation in response to various stimuli and abnormalities. **c** CxI, CxII and CxIV activities in whole cochlear extracts from WT (blue bars) and p43^−/−^ (red bars) mice aged 1 and 10 months. DCIP: 2,6-dichloroindophenolate. Data are expressed as mean ± SD (*n* = 8 mice per age and strain). One-way ANOVA was followed by *Dunn’s* test (*P = 0.037, **P = 0.004, p43^−/−^ vs. WT of the same age; *P = 0.037, ***P ≤ 0.001, older p43^−/−^ vs. 1-month-old p43^−/−^; *P ≤ 0.05, older WT vs. 1-month-old WT). **d**–**f** Confocal images of transverse cryostat sections of the organ of Corti (**d**) and SGNs (**e**, **f**) from WT and p43^−/−^ mice at 1 and 6 months. The sections were immunolabeled for cytochrome c oxidase (green), Myosin 7A (red), and Hoechst (blue). Reduction of cytochrome c oxidase was only observed in the SGNs, hair cells, and cochlear nerve fibers of 6-month-old p43^−/−^ mice. The arrowheads in **d** indicate the cochlear nerve fibers. DCs: Deiters cells. Scale bars: **a** = 0.5 μm, **d**–**f** = 20 μm. **g** Quantitative analysis of malondialdehyde (MDA) in whole cochlear extracts from WT (blue bars) and p43^−/−^ (red bars) mice aged 1 and 10 months of age. Data are expressed as mean ± SD (*n* = 8 mice per age and strain). One-way ANOVA test was followed by *Dunn’s* test (***P = 0.001, p43^−/−^ vs. WT of the same age; ***P ≤ 0.001, older p43^−/−^ vs. 1-month-old p43^−/−^; *P = 0.04, older WT vs. 1-month-old WT). **h** Representative western blot (Inset) and histograms of SIRT1 in whole cochlear extracts from WT (blue bars) and p43^−/−^ (red bars) mice aged 1, 6, and 12 months. **i**: Representative western blot analysis using antibodies against Foxo3a, LC3B, Rab7, BNIP3, and β-actin in whole cochlear extracts from WT and p43^−/−^ mice aged 1, 3, 6, and 12 months. **j**, **k**: Histograms representing the levels of Foxo3a, LC3-II, Rab7, and BNIP3 in WT (blue bars) and p43^−/−^ (red bars). β-actin served as a loading control. Data are expressed as mean ± SD (*n* = 24 cochleae per age and strain. Each experiment was performed with a pool of 8 cochleae per sample, and in biological and technical triplicate). One-way ANOVA test was followed by *Dunn’s* test (*P ≤ 0.05, ** *P* ≤ 0.01, *** *P* ≤ 0.001, p43^−/−^ vs. WT of the same age; * *P* ≤ 0.05, ***P ≤ 0.001, older p43^−/−^ vs. 1-month-old p43^−/−^; *P ≤ 0.05, older WT vs. 1-month-old WT)

One characteristic feature of p43 deletion in the cochlear sensory hair cells is the presence of an increased size of mitochondria, and mitochondria with altered cristae and matrix (Fig. 5a). Measurements revealed a significant increase in the mitochondrial diameter in both IHCs and OHCs of KO mice at 1 month, compared with those in WT mice of the same age (*P* ≤ 0.001, Fig. 5b). In the subsequent months, WT mice developed a progressive increase in the mitochondrial diameter, while p43^−/−^ mice significantly decreased theirs (*P* ≤ 0.001, Fig. 5b). However, the mitochondrial diameters in KO mice were significantly larger than those in WT at all ages analyzed (*P* ≤ 0.05, Fig. 5b), except in the OHCs of KO mice aged 12 months.

To investigate the mechanisms underlying the changes in mitochondria, we compared the enzymatic activity of respiratory chain complexes I (CxI), II (CxII), and cytochrome c oxidase (CxIV or COX, insert Fig. 5c), between p43^−/−^ and WT mice aged 1 and 10 months. Our results showed no significant difference in the enzymatic activity of mitochondrial complexes between p43^−/−^ and WT mice at 1-month age. At 10 months of age, however, a significant increase (*P* ≤ 0.05 versus 1 month) of CxI was observed in WT mice, whereas we found a strong decrease (*P* ≤ 0.05 versus 1 month) of this activity in p43^−/−^ mice, leading to a significantly lower level of CxI activity in KO mice than in WT (*P* ≤ 0.01, Fig. 5c). In contrast, complex II activity was reduced in both strains at 10 months of age (WT: *P* ≤ 0.05, p43^−/−^: *P* ≤ 0.001), making it unlikely to fully account for the phenotype associated with p43 deletion. Finally, a significantly lower level of CxIV activity was also observed in p43^−/−^ compared to WT mice aged 10 months (*P* ≤ 0.05).

The reduced CxIV activity was confirmed by confocal microscopy analysis. One-month-old p43^−/−^ and WT cochleae exhibited intense and punctuated COX staining, mainly in the cytoplasm of sensory hair cells and SGNs, but also in auditory nerve fibers under the sensory hair cells (Fig. 5d–f). At 6 months, COX labeling decreased only in p43^−/−^ mice (Fig. 5d, f).

In addition, a significantly increased level of MDA, a lipid peroxidation marker, was observed with aging in both WT (*P* ≤ 0.05) and p43^−/−^ mice (*P* ≤ 0.001). By 10 months, however, MDA accumulation was much higher in p43^−/−^ than in WT mice (*P* ≤ 0.001, Fig. 5g).

Sirtuin 1 (SIRT1) is a NAD+-dependent deacetylase involved in aging and metabolic regulation [26]. Western-blot analyses revealed a drastic fall of SIRT1 expression in p43^−/−^ compared to the WT mice at 1 and 12 months of age (*P* ≤ 0.01 vs. WT mice of the same age, Fig. 5h). In addition, a significant increase in SIRT1 levels with age was seen in WT mice at 12 months (*P* ≤ 0.01 vs. 1 month, Fig. 5h) and in p43^−/−^ from 6 months and maintained to 12 months (*P* ≤ 0.01 vs. 1 month, Fig. 5h).

Altogether, these results show that p43 deletion was associated with altered mitochondrial morphology and function, oxidative stress, and reduced expression of SIRT1.

### Upregulation of autophagy in p43^−/−^ mice

Considering the link between oxidative stress, mitophagy, and autophagy [27], we examined autophagy induction by monitoring the levels of proteins involved in the autophagic process. Foxo3a is a forkhead transcription factor of the Foxo class that plays an important role in regulation of autophagy. Here, we found that p43^−/−^ mouse cochleae displayed a strong increase in the levels of Foxo3a (*P* ≤ 0.01) from 3 months of age and maintained this to 12 months, compared with WT mice of the same age (Fig. 5i-j). Correspondingly, a higher accumulation of the lipid form of LC3B, a hallmark of autophagosome formation, was observed in p43^−/−^ mice from 6 months and maintained to 12 months (Fig. 5i, j, *P* ≤ 0.05). Immunofluorescence experiments shed more light on the cells concerned. Thus, a similar level and a diffuse and uniform pattern of LC3B staining was observed in the cytoplasm of the sensory hair cells and supporting cells of the organ of Corti in 1- and 6-month-old WT (Additional file 1: Fig. S3A, E) and p43^−/−^ cochleae (Additional file 1: Fig. S3C, G). By contrast, a punctuate LC3B staining was observed as early as 1 month in SGNs of both strains (Additional file 1: Fig. S3B, D, I and K). At 6 months, this punctuate staining strongly increased in p43^−/−^ mice (Additional file 1: Fig. S3H and L), but not in WT mice (Additional file 1: Fig. S3F and J) suggesting the increased formation of autophagosome in the SGNs of KO mice. Rab7 is a small, GTP-binding protein playing a role in the maturation of late autophagic vacuoles. We observed a significant age-related increase of Rab7 in both WT (*P* ≤ 0.05) and KO (*P* ≤ 0.001) mice aged 12 months (Fig. 5i, k).

BNIP3 is a pro-apoptotic protein related to the BH3-only family, which induces both cell death and autophagy [28]. Our results showed similar expression levels of BNIP3 in both strains from 1 to 6 months of age. However, at 12 months, we observed a two-fold increase of BNIP3 in p43^−/−^ mice, compared to WT mice (*P* ≤ 0.001, Fig. 5i, k). Taken together, these results indicate that p43 deletion induced an increased autophagic response, mainly in the SGNs.

### Macrophage invasion and increased inflammatory protein expression in p43^−/−^ mice

**Fig. 6:**
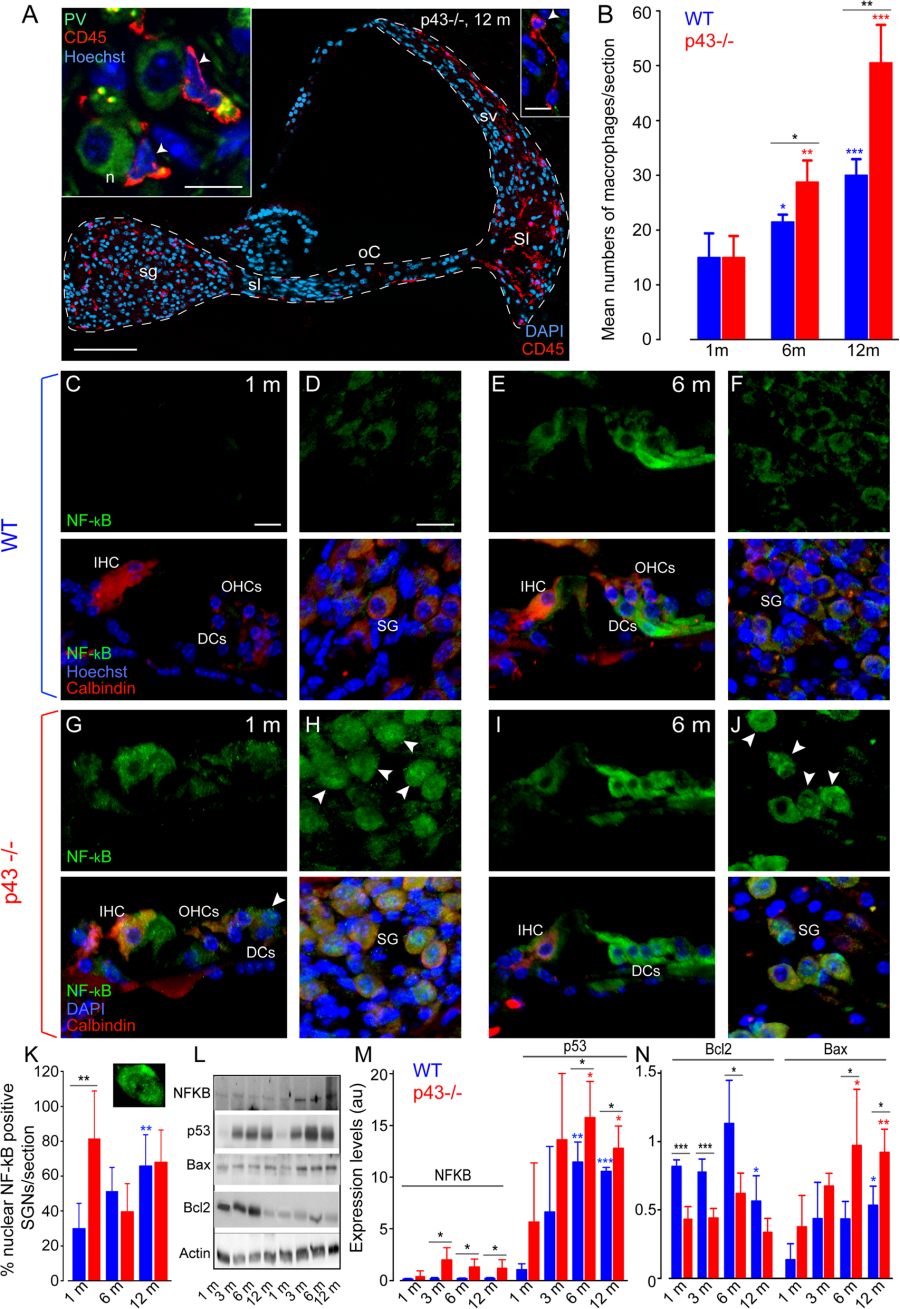
Macrophage invasion, pro-apoptotic protein expression. **a**: confocal images of transverse cryostat sections of the cochleae from p43^−/−^ mice at 12 months. The sections were immunolabeled for CD45 (red), allowing macrophage detection and for parvalbumin (PV, green) to identify the SGNs, and counter-stained with Hoechst to label nuclear chromatin (blue). Note dramatic invasion of CD45 positive macrophages in the spiral ganglion (sg), the spiral lamina (sl), the organ of Corti (oC), the spiral ligament (Sl), and the stria vascularis (sv) of 12-month-old p43^−/−^. The left inset shows macrophages (red) surrounding the SGNs and the right inset, those in the spiral ligament. White arrowheads indicate macrophages. Scale bars: **a** = 100 μm, left inset = 20 μm, right inset = 10 μm. n, neuron. **b** Histogram representing the average number of macrophages per section from the transverse sections of the cochlear basal region of both WT (blue bars) and p43^−/−^ (red bars) aged 1, 6, and 12 months. Data are expressed as mean ± SD (*n* = 4 or 5 sections per cochlea, 4 cochleae per age and strain). One-way ANOVA test was followed by *Dunn’s* test (*P ≤ 0.05, **P ≤ 0.01, p43^−/−^ vs. WT of the same age; **P ≤ 0.01, ***P ≤ 0.001, older p43^−/−^ vs. 1-month-old p43^−/−^; *P ≤ 0.05, ***P ≤ 0.001, older WT vs. 1-month-old WT). **c**–**j** Confocal images of transverse cryostat sections of the organ of Corti (**c**, **e**, **g**, and **i**), and spiral ganglion (SG, **d**, **f**, **h**, and **j**) at 1 (**c**, **d**, **g**, and **h**) and 6 months (**e**, **f**, **i**, and **j**) from WT and p43^−/−^ mice. The sections were labeled for NF-κB (green), and for calbindin (red) to identify the hair cells and SGNs, and counter-stained with Hoechst dye (blue). Note massive nuclear translocation of NF-κB in the SGNs of p43^*−/−*^ mice aged 1 month (**h**). White arrowheads indicate nuclear NF-κB-positive SGNs. Scale bars: **c**, **g**, **e**, **i** = 10 μm, **d**, **h**, **f**, **j** = 20 μm. DCs, Deiters cells. **k** Histogram representing the percentage of nuclear NFκB-positive SGNs per section from the transverse sections of the cochlear basal regions of both WT (blue bars) and p43^−/−^ (red bars) aged 1, 6, and 12 months. Data are expressed as mean ± SD (*n* = 4 or 5 sections per cochlea, 5 cochleae per age and strain). One-way ANOVA test was followed by *Dunn’s test* (**P ≤ 0.01, p43^−/−^ vs. WT of the same age; **P ≤ 0.01, older WT vs. 1-month-old WT). **l** Representative western blot analysis using antibodies against NF-κB, p53, Bax, Bcl2 and β-actin in whole cochlear extracts from WT and p43^−/−^ mice aged 1, 3, 6, and 12 months. **m**, **n** Histograms representing the levels of NF-κB*, p53,* Bcl2 and Bax in WT (blue bars) and p43^−/−^ (red bars). β-actin served as a loading control. Data are expressed as mean ± SD (each experiment was performed with a pool of 8 cochleae per sample per age and per strain, and in biological and technical triplicate). One-way ANOVA test was followed by *Dunn’s* test (*P ≤ 0.05, ***P ≤ 0.001, p43^−/−^ vs. WT of the same age; *P ≤ 0.05, **P ≤ 0.01, older p43^−/−^ vs. 1-month-old p43^−/−^; *P ≤ 0.05, **P ≤ 0.01, ***P ≤ 0.001, older WT vs. 1-month-old WT)

The disappearance of cells is accompanied by an immune system response characterized by an invasion of many macrophages. To identify the macrophages, we used CD45, a pan-leukocyte marker used to visualize immune cells. We had already shown that 90% of CD45-labeled leukocytes present in the amikacin-damaged cochlea were macrophages [29]. Here, we found an invasion of CD45-positive macrophages in the spiral ganglion, organ of Corti, and spiral ligament (Fig. 6a) of the cochleae of both strains during aging. A significant increase in the numbers of macrophages was seen in both strains from 6 months of age and maintained to 12 months (6 months: WT: *P* ≤ 0.05, KO: *P* ≤ 0.01; 12 months: WT and KO: P ≤ 0.001, versus 1 month). However, we found that from 6 months of age, the numbers of macrophages increased more strongly in p43^−/−^ mice than in WT mice (*P* ≤ 0.05, Fig. 6b).

The dramatic increase in macrophages led us to probe the expression and localization of NF-κB, a transcription factor regulating multiple aspects of innate and adaptive immune functions and serving as a pivotal mediator of inflammatory responses [30]. Our results showed an early nuclear translocation of NF-κB, mainly in the spiral ganglion neurons, and occasionally in a few supporting cells of the organ of Corti of 1-month-old p43^−/−^ mice (Fig. 6g, h) compared with WT mice of the same age (Fig. 6c, d). At 6 months of age, nuclear translocation of NF-κB was only observed in spiral ganglion neurons of both strains (Fig. 6f, j), but not in the cells of organ of Corti (Fig. 6e, i). Counting nuclear NF-κB-positive SGNs demonstrated a significant nuclear translocation of NF-κB in SGNs of p43^−/−^ mice at 1 month of age compared with WT mice of the same age (*P* ≤ 0.01, Fig. 6k). While an age-related increase of nuclear NFκB-positive SGNs was observed in WT mice only at 12 months (*P* ≤ 0.01 vs. 1 month, Fig. 6k), high numbers of nuclear NF-κB-positive SGNs were maintained to 12-month-old in p43^−/−^ mice (Fig. 6k). Consistent with an early nuclear translocation of NF-κB in the SGNs of p43^−/−^ mice, a strong increase in the levels of NF-κB was observed in the cochlear tissues of p43^−/−^ mice from 3 to 12 months of age (*P* ≤ 0.05) compared with WT mice of the same ages (Fig. 6l, m).

Altogether, these data clearly demonstrate the occurrence of an inflammatory phenotype in p43^−/−^ mouse cochleae.

### Upregulation of pro-apoptotic protein in p43^−/−^ mice

p53 is a transcription factor that plays an important role in modulating distinct cell-fate decisions [31]. Here, we showed a significantly increased level of p53 expression in both strains during aging when compared to animals 1 month of age, but with significantly higher levels in the cochleae of p43^−/−^ mice compared to WT mice from 6 to 12 months (*P* ≤ 0.05, Fig. 6l, m). Interestingly, we observed that the expression of the pro-survival Bcl2 protein is halved in p43^−/−^ mice, whatever their age, compared to controls (1 and 3 months: *P* ≤ 0.001; 6 months: *P* ≤ 0.05 vs. WT of the same age, Fig. 6l, n). By contrast, an age-related reduction of Bcl2 protein was seen only in WT mice at 12 months (*P* ≤ 0.05 vs. 1 month, Fig. 6n). Conversely, the levels of pro-apoptotic Bax protein were increased in both strains during aging, but earlier in p43^−/−^ than in WT mice (6 versus 12 months for KO and WT mice, respectively, Fig. 6l, n). In addition, Bax expression levels were significantly higher in p43^−/−^ mice cochleae than in WT mice from 6 to 12 months of age (*P* ≤ 0.05 vs. WT of the same age, Fig. 6n). Together, these results suggest that p43 deletion enhanced age-related activation of cochlear-cell apoptosis in mice.

### P43 deletion enhances noise-induced hearing loss

It is well known that mouse strains exhibiting enhanced ARHL are also more sensitive to noise-induced hearing loss (NIHL) than are other strains [26]. We thus probed the effect of p43 deletion on NIHL. Our results showed that ABR thresholds were almost the same between the two genotypes before acoustic overstimulation (Additional file 1: Fig. S4A-B). The overstimulation induced similar temporary threshold shifts (TTS) in both strains (Additional file 1: Fig. S4A-C). By contrast, threshold recovery was significantly better in WT than in p43^−/−^ mice at all frequencies tested 2 weeks after the acoustic trauma (*P* ≤ 0.01, Additional file 1: Fig. S4A-B, D). These results suggest that p43^−/−^ animals were less capable of recovering from NIHL than were controls.

## Discussion

We report here, for the first time, that p43 deletion enhanced age-related hearing loss in mice. Exacerbated ARHL was associated with early-reduced, pro-survival protein expression, a decrease of mitochondrial respiratory chain activities, together with an increase of pro-inflammatory protein, autophagy, and pro-apoptotic protein expression, leading to enhanced degeneration of OHCs, SGNs, and IHCs. The fact that SGN loss occurred before IHC death indicates that degeneration of SGNs is not a consequence of IHC loss. Not unexpectedly, p43-deficient mice were also more susceptible to noise damage than WT mice.

### Exacerbated age-related hearing loss

The mice used in this study are a C57BL/6 J background strain that is commonly used in transgenic and knockout production. This strain carrying the *Cdh23*^*ahl*^ mutation is known to exhibit a moderate high-frequency hearing loss by 6 months of age that progresses to almost complete high-frequency deafness by 18 months [32, 33]. Here, WT mice displayed a similar classic pattern of ARHL, which is consistent with previous reports [32, 33]. By contrast, more severe age-related elevation of the ABR thresholds and greater reduction of DPOAE was observed in p43^−/−^ mice than in WT mice. Thus, the profound DPOAE reductions observed in p43-deficient mice, indicating OHC dysfunction, account, at least in part, for the bulk of the threshold elevation in mutant mice. In addition, stria vascularis dysfunction is unlikely to be an important contributor to deafness, since the endocochlear potential was preserved in p43^−/−^ until 12 months of age. The elevation of ABR thresholds and reduction of DPOAE amplitudes are similar from 1 to 6 months of age in p43^−/−^. By contrast, the reduction of DPOAE amplitudes were smaller than the elevation of ABR thresholds at 12 months, which suggests that, in addition to possible differences originating in OHCs that would affect the DPOAE thresholds, there are also changes originating in IHCs and SGNs that would produce additional effects on the ABR responses in p43^−/−^ mice.

The exacerbated ARHL in p43 mice may reflect failure of homeostatic functions at adult stages. Another scenario could be that subtle defects that may compromise the long-term maintenance of cochlear function accumulate in immature p43^−/−^ mice, and these may have greater vulnerability to lifelong exposure to risk factors of ARHL [34]. Loss-of-function mutations in the gene encoding the SLC7A8 protein, an energy-independent exchanger of neutral amino acids and thyroid hormone transporter, are associated with human ARHL [35]. It will be interesting to probe potential changes in the thyroid hormone transporters in the absence of p43 by further in-depth investigations.

### Enhanced age-related loss of hair cells

In p43^−/−^ mice at 11 days after birth, the overall organization of the organ of Corti developed normally. By contrast, more severe age-related death of OHCs and, to a lesser extent of IHCs, was observed in p43^−/−^ mice. The greater age-related OHC loss in the p43 strain is likely to account for at least part of the enhanced ARHL, because the timing is similar, and the fraction of OHC deaths is quite substantial. Previous reports demonstrated the loss of OHCs in some models of both congenital hypothyroidism [36] and TRβ^−/−^ mice [37]. Ng et al. [12] reported that TRβ1 is required for the long-term survival of hair cells during cochlear aging, and here we showed for the first time that p43, a truncated form of TRα1, is required for the long-term survival of the OHCs, and to a lesser degree of IHCs. A previous study demonstrated that OHC system is the most susceptible to the developmental effects of congenital hypothyroidism [38], but the progressive hearing impairment characteristic of p43^−/−^ may result from compounding effects of subtle defects of hormone-dependent processes in immature p43^−/−^ mice, together with cochlear cell stress in the absence of p43 in adult mice.

### Early-onset and progressive myelin damage and SGN loss

In the mouse cochlea, myelination by Schwann cells starts near birth at the cell body of SGNs and progresses to the central and peripheral processes between postnatal days 8 and 10, just before the onset of hearing [39]. It has been proposed that TRβ is necessary for myelinogenesis of the auditory nerve [40]. In the present study, ultrastructural pathological changes in myelin and glial cells were already observed in p43^−/−^ mice aged 1 month. In addition, a dramatic reduction in MBP expression was observed early, and more severely, in KO mice compared with WT mice. Based on the important roles of SGN-associated glial cells in myelin formation, maintaining homeostasis, and in providing neurotrophic support to the auditory nerve through a reciprocal signaling mechanism [41], it is no surprise to also see a significant exacerbation of age-related SGN loss in p43^−/−^ mice from 6 months of age. Our results illustrate the susceptibility of cochlear myelin to age-related damage in the absence of p43 in mice. These findings are in agreement with other reports demonstrating that thyroid hormone may promote myelin repair and adult neurogenesis via endogenous stem and precursor cells in the central nervous system [42, 43].

### Alteration of mitochondrial morphology and function, and reduced SIRT1 expression

Mitochondria are dynamic organelles whose morphology is directly linked to the maintenance of their functions [44], and the disruption of their normal shape is a hallmark of mitochondrial dysfunction. Here, we found the early occurrence of enlarged mitochondria, mainly in the sensory hair cells. These enlarged mitochondria may reflect an attempt to compensate for the deficit of activity of the respiratory chain, as has often been observed in aging and cellular senescence [45]. In addition, mitochondria with alteration of their cristae and matrix were also observed in the cochlear sensory neural cells of p43^−/−^ mice.

In line with the alteration of mitochondrial morphology, we demonstrated that p43 deletion induced a decreased activity of CxI and CxIV of the mitochondrial respiratory chain activities in cochlear tissues. This is in agreement with previous studies showing alterations of respiratory chain activities in p43-depleted tissues such as skeletal muscle and pancreatic islets [46]. Since p43 is a T3-dependent transcription factor of the mitochondrial genome, its absence impaired the expression of subunits of these complexes encoded by that genome. Consistent with this, the activity of CxII, including only subunits encoded by nuclear genes, is not affected by p43 depletion. Taken together, our results suggest that p43 plays a non-negligible role in the maintenance of mitochondrial function and morphology in the cochlear sensory neural cells in adulthood.

SIRT1, a major deacetylase sensitive to the cellular redox state, plays diverse roles in gene silencing and stress resistance [47]. A reduction of SIRT1 leads to the loss of its control on acetylation of target proteins, including p53, NF-κB, and FOXO3, thereby enhancing the inflammatory, prosenescent, and apoptotic responses [47]. Here, we observed an increase of SIRT1 expression in the cochleae of both strains during aging that may be a compensatory mechanism for the accumulation of oxidative stress-related products and the reduction of antioxidant enzyme levels during aging, as seen in older humans [48]. Interestingly, an early, drastic fall of SIRT1 expression was observed in 1-month-old p43^−/−^ mice, and this was maintained until 12 months of age. The drastic reduction of SIRT1 may result from a mitochondrial complex I deficiency, as showed in Charcot-Marie-Tooth disease type 2K [49], or from failed mitochondrial/nuclear crosstalk in the absence of p43 (Additional file 1: Fig. S5) [18]. Impaired SIRT1 expression observed in the cochlear cells of p43^−/−^ may play a critical role in the increased susceptibility of the cochlea to aging, as reported for the heart [50].

### Upregulation of autophagy

Here, we showed that p43^−/−^ mice exhibited increased activity of autophagy, as illustrated by the increased expression, of several autophagy-related proteins, such as FOXO3a, which have been shown to increase transcription of autophagy-related genes and stimulate autophagy [51]. In addition, we also observed increased levels of LC3B-II, Rab7, and BNIP3 in the cochlear tissues as well as the accumulation of autophagic vehicles in cochlear sensory-neural cells of p43^−/−^ mice during aging. Consistent with previous studies demonstrating that autophagy helps maintain adult hearing in response to cochlear stress induced by ototoxic drugs, noise, and aging [52–54], our results demonstrate that p43 deletion triggers an increased autophagic response, which may reflect a pro-survival function to remove damaged mitochondria and to respond to auditory cell stress in the absence of p43.

### Oxidative stress, chronic inflammation, and increased pro-apoptotic protein expression

Here, we observed that oxidative stress was strongly induced during aging, as demonstrated by the overproduction of MDA in p43 ^−/−^ mice. As expected, p43^−/−^ cochleae also exhibited an abundance of macrophages, with a concomitant increase of the expression level and early nuclear translocation of NF-κB. Indeed, inflammation and oxidative stress are two closely related processes that are linked to thyroid hormone disturbance in a reciprocal manner [55]. It has been reported that T3 plays a role in the control of macrophage maturation and functions through TRβ2 receptor [56]. In addition, ligand-bound TRα on macrophages plays a protective role in kidney inflammation through the inhibition of NF-κB pathways [57]. Our findings provide evidence that the p43 deficiency exacerbated oxidative stress and inflammatory processes in cochlear tissues in adulthood.

We then evaluated the expression patterns of pro-apoptotic BAX, anti-apoptotic Bcl-2, and p53, the proposed upstream effector of these molecules, which play a key role in the inhibition of intrinsic apoptotic pathways that are triggered by mitochondrial dysfunction. We showed a significantly reduced level of Bcl-2, with a concomitant increase of Bax and p53 expression, in p43^−/−^ mice, suggesting an imbalance between pro- and anti-apoptotic members in the cochleae of p43^−/−^.

## Conclusion

Our data indicate that mice lacking p43 were more vulnerable to age-related and noise-induced hearing loss. These findings demonstrate that p43, by regulating mitochondrial activity, plays a beneficial role in the long-term maintenance of cochlear homeostasis and cell function against lifelong exposure to sound and other risk factors of ARHL in mice. The deletion of p43 leads to the alteration of mitochondrial morphology and function, a drastic fall of SIRT1 and Bcl2 expression, and a subsequent increase of oxidative stress, inflammation, and apoptosis, together leading to enhanced ARHL and sensory-neural cell death (Additional file 1: Fig. S5). Our data provide evidence that TRα is required for the maintenance of hearing in adulthood, and suggest that human *THRA* gene polymorphisms and/or mutations may be responsible for some adult-onset progressive deafness or syndromic deafness.

## Methods

This study is designed to explore the role of p43 in hearing function and in the maintenance of hearing during aging in p43^−/−^ mice through complementary approaches combining morpho-physiology, biochemistry and molecular biology.

### Animals

The p43^−/−^ mice were generated by back-crossing more than 10 times into the C57bl/6 background [22]. We generated our colony by crossing p43^−/−^mice with wild-type C57bl/6 breeders and generated future generations of wild-type controls (WT). In this study, to avoid interfering results from the protective effect of estrogen of female mice against ARHL, we only used male mice. Mice used in the present study were the result of homozygous matings; therefore, no heterozygotes were tested. However, the mice were the offspring of six KO mating pairs and four WT pairs formed from the progeny of a single heterozygous mating pair, so the contributions of the genetic modifiers were randomized. The mice were housed in pathogen-free animal-care facilities accredited by the French Ministry of Agriculture and Forestry (C-34-172-36; December 19, 2014). Experiments were carried out in accordance with French Ethical Committee stipulations regarding the care and use of animals for experimental procedures (agreements C75-05-18 and 01476.02, license #6711).

### Genotyping

Genotyping was performed using routine PCR with the following primer sequences (forward and reverse, respectively): p43 KO (5′-CCC TTG CTG TGA CAC TCG TAG CT-3′ and: 5′-TGA CAG CGC TAG GCA CTG-3′). All primers were synthesized by Eurofins MWG Operon.

### Noise exposure

We used an 8- to 16-kHz-band noise at 100 dB SPL for 2 h, adapted from Kujawa and Liberman [58]. Hearing is most sensitive for mice at frequencies of approximately 16 kHz. We adjusted the levels of the noise in a pilot study to obtain elevated ABR thresholds in WT mice 1 h following noise exposure, and returning to nearly normal 15 days after exposure. Awake 1-month-old p43^−/−^ male mice and their WT were placed unrestrained in a subdivided cage with one mouse per division. The noise was generated by a PCI 4461 card (National instruments) controlled by LabVIEW software. The sound level was calibrated before each exposure session using a 1/4 in. microphone (# 46BE, GRAS Sound & Vibration) controlled by a PCI 4461 card and LabVIEW so that there was no more than a 1 dB difference between the center and the edge of the cage.

### Functional hearing assessments

All functional evaluations were performed under anesthesia. Fifteen mice of each strain were successful monitored by auditory brainstem response (ABR) and distortion product otoacoustic emission (DPOAE) assessments up to 12 months of age. Among them, 8 were randomly selected at 12 months of age for EP recording and sacrificed for cochlear morphological assessments, seven were followed up to 18 months. Endocochlear potential (EP) recording required 16 additional animals aged 1 and 6 months (*n* = 8 per age and per strain). After ABR, DPOAE, and EP recording, their cochleae were removed for morphological evaluation. For evaluation of noise-induced hearing loss, 30 additional 1-month-old mice (*n* = 15 per strain) were recorded before, 1 h and 2 weeks after noise exposure. All functional evaluations were carried out in a Faraday-shielded, anechoic, sound-proof cage. Rectal temperature was measured with a thermistor probe and maintained at 38.5 °C ± 1 using an underlying, heated blanket.

#### DPOAEs

DPOAEs were recorded in the external auditory canal using an ER-10C S/N 2528 probe (Etymotic research Inc. Elk Grove Village, IL, USA). The two primary tones of frequency f1 and f2 with a constant f2/f1 ratio of 1.2 were generated, and the distortion product 2f1-f2 processed, by a Cubdis system HID 40133DP (Mimosa Acoustics Inc., Champaign, IL, USA). The probe was self-calibrated for the two stimulating tones before each recording. f1 and f2 were presented simultaneously, sweeping f2 from 20 to 2 kHz by quarter octave steps. For each frequency, the distortion product 2f1-f2 and the neighboring noise amplitude levels were measured and expressed as a function of f2.

#### ABRs

ABRs were recorded using three subcutaneous needle electrodes placed on the vertex (active), on the pinna of the tested ear (reference), and in the hind leg (ground). Strong correlations were observed between click-evoked ABR thresholds and pure-tone thresholds at 2 and 4 kHz [59]. To obtain more frequency-specific estimates of hearing sensitivity in the high-frequency range, we chose to use tone-burst stimulation for ABR recording. Sound stimuli were generated by a NI PXI-4461 signal generator (National Instruments) and consisted of 10 ms tone-bursts with a 1 ms rise- and fall time, delivered at a rate of 10/s. Sound was produced by a JBL 075 loudspeaker (James B. Lansing Sound) positioned at 10 cm from the tested ear in a calibrated, free-field condition. Cochlear-responses were amplified (20,000) via a Grass P511 differential amplifier, and averaged 1000 times (Dell Dimensions). Intensity-amplitude functions of the ABRs were obtained at each frequency tested (2, 4, 6.3, 8, 10, 12.5, 16, 20, 24, and 32 kHz) by varying the level of the tone bursts from 0 to 100 dB SPL, in 5 dB incremental steps. The ABR thresholds were defined as the minimum sound intensity necessary to elicit well-defined and reproducible wave II. Recordings and analysis were performed blindly.

#### Endocochlear potential (EP)

To measure the EP, the bone of the scala media basal turn was gently shaved off, resulting in a small fenestra. A glass microelectrode (tip diameter 0.1–0.5 μm), filled with 0.15 M KCl and connected to a direct current amplifier (WPI, model 773 A; Sarasota, FL, USA), was placed visually at a position and angle allowing it to pass through the fenestra to record the EP with reference to an Ag/AgCl reference electrode in the neck musculature of the animal.

### Morphological assessments

Gross examination of outer- and middle-ear structures did not identify any visible structural changes in p43^−/−^ mice compared to WT mice at 1 month of age. The ultrastructural characteristics of the cochlear sensory neural cells in p11 and 1-, 6-, and 12-month-old mice were analyzed using scanning electron microscopy (SEM, Hitachi S4000) and transmission electron microscopy (TEM, Hitachi 7100).

#### Counting of sensory hair cells

Sensory hair-cell loss was evaluated using SEM. The cochleae were processed and evaluated using previously reported, standard techniques [29]. Counting of inner (IHC) and outer (OHC) hair cells was performed in the apical (0.5 to 1 mm from apex tip, corresponding to the 6 to 8 kHz region), mid (1.9 to 3.3 mm from the apex tip, corresponding to the 12 to 24 kHz region), and basal (4.1 to 5.0 mm from the apex tip, corresponding to 32 to 50 kHz region) regions of the cochlea (*n* = 6 to 12 mice per age and strain). Hair cells were considered to be absent if the stereociliary bundles and cuticular plates were missing.

#### Counting of spiral ganglion neurons

The spiral ganglion neuron (SGN) density in Rosenthal’s canal was measured using a Zeiss Axioskop light microscope in semi-thin sections that had been cut during the course of TEM preparation and stained with 1% toluidine blue. The SGN counts were calculated in the basal region of the cochlea. NIH Image J software was used to determine the cross-sectional area of Rosenthal’s canal. SGN density was calculated by dividing the number of neurons by the cross-sectional area (*n* = five sections per cochlea, 7 to 9 cochleae per age and strain).

#### Ultrastructural analysis

Morphological damage related to p43 deletion was investigated using TEM investigations focused on the basal cochlear region. Animals were decapitated under deep anesthesia, and their cochleae were prepared according to a standard protocol for fixation and plastic embedding. Semi-thin sections were observed under a Zeiss Axioskop light microscope, and ultrathin radial sections of the organ of Corti were observed using TEM (*n* = 4 cochleae per age and strain).

#### Measure of mitochondrial diameter

The diameters of mitochondria were measured on TEM micrographs using the length measurement software (TIA) of the TEM, which provided the value in nm of the distance between two points previously positioned on the external membrane of the mitochondria by the observer. The vast majority of mitochondria selected for diameter measurement were more-or-less round, and the two points were positioned along the larger axis of the mitochondria. For each cochlea, ~ 45 to50 mitochondria, taken randomly from the IHCs or OHCs (*n* = 4 cochleae per age and strain), were used for diameter measurements.

#### Age-related hearing impairment and morphological correlation

To compare the time course of the hearing impairments found in p43^−/−^ and WT mice, the mean ABR threshold evoked by tone-bursts of frequencies 16 kHz, and the mean DPOAE obtained at an *f2 frequency* of 16 kHz, were calculated for each strain and at each time point. A linear regression was then performed to determine the threshold elevation and DPOAE amplitude decrease per month. To decipher the contribution of the reduced DPOAE amplitude to the accelerated age-related hearing impairment in p43 KO mice, we used the mean values of ABR thresholds and DPOAE amplitude in WT mice as a reference and subtracted these from the values for KO mice. The differences in threshold and DPOAE (p43^−/−^ minus mean WT) were then averaged and used for statistical analysis. Percentages of remaining OHCs, IHCs, and SGNs observed in WT and p43^−/−^ mice at each time point normalized to those in 1-month-old WT mice, which were put at 100%.

### Molecular assessment

#### Enzymatic activities and lipid peroxidation

Cochlear homogenates were prepared as described by Casas [60]. The protein concentration was measured using the Bradford method. Complex (Cx) I, II and cytochrome oxidase activities were measured as previously described [60] and expressed in mU/mg protein. Lipid peroxidation was assessed using the thiobarbituric acid-reactive substances method, and was expressed in nmol/mg malondialdehyde (MDA) [60]. Enzymatic activities and lipid-peroxidation analysis required 8 additional animals (16 cochleae) per age and strain. All experiments were performed in triplicate.

#### Western blotting

Cochlear homogenates were prepared in Laemmli sample buffer. Blots were incubated with antibodies recognizing Sirtuin 1 (1/500, Cell Signaling # 8469 RRID:AB-10999470), FOXO3a (1/1000, Cell Signaling #2497 RRID:AB-836876), LC3B (1/800, Cell Signaling #2775 RRID:AB-915950), Rab7 (1/800, Santa Cruz Biotechnology #sc-376362 RRID:AB-10987863), BNIP3 (1/1000, Abcam, #Ab10433 RRID:AB-2066656), p53 (1/1500, Cell Signaling Technology, #2524 RRID:AB-331743), Bax (1/1000, Abcam #7977 RRID:AB-306191) and BCl2 (1/1000, Santa Cruz #sc-492 RRID:AB-2064290). β-actin (1/10000, Sigma-Aldrich #A1978 RRID:AB-476692) served as a loading control. The secondary antibodies used were horseradish peroxidase-conjugated goat anti-mouse IgG (1/3000, Jackson ImmunoResearch #115-001-003 RRID:AB-2338443), or goat anti-rabbit IgG (1/3000, Jackson ImmunoResearch #111-001-003 RRID:AB-2337910). Image scans of Western blots were used for semi-quantitative analysis. Western blot analysis required 12 additional animals (24 cochleae) per age and strain. Each experiment with a pool of 8 cochleae (4 animals per samples) was performed in biological and technical triplicate.

#### Immunocytochemistry

Immunocytochemistry was employed to probe *myelin* protein abundance, thyroid hormone receptor alpha as well as the cellular localization of some cell survival, autophagic, and pro-inflammatory markers in cryostat sections using antibodies recognizing myelin basic protein (MBP, 1/200, Santa Cruz Biotechnology, sc-271524 RRID:AB-10655672), thyroid hormone receptor alpha 1,2 (1/100 dilution, Thermo Fisher, #PA1-21134 RRID: AB_561695), Cytochrome c oxidase subunit I (1/500 dilution; Invitrogen, #459600 RRID:AB-1501840), LC3B (1/500, Cell Signaling #2775 RRID:AB-915950), and CD45 (1/100, Merck Millipore, #05-1410), NF-κB (1/400, p65 subunit, Millipore, #Mab3026 RRID:AB-2178887). Anti-parvalbumin (1/500; Swant, Bellinzona, Switzerland, #PV235) and anti-calbindin-D-28 K (1:200 dilution; Sigma-Aldrich, #C9848 RRID:AB-476894) were used to label the hair cells and the spiral ganglion neurons. Anti-myosin 7A (1/300, Proteus Biosciences Inc #25-6790 RRID:AB-10015251) and anti-neurofilament (NF 200, 1/600, Sigma-Aldrich #N0142 RRID:AB-477257) were used to identify the hair cells and spiral ganglion neurons, respectively. All secondary antibodies were used at a dilution of 1/1000. This included donkey anti-mouse and anti-rabbit IgG conjugated to Alexa 488 or Alexa 568 (Molecular Probes #A-21202 RRID:AB-141607, #A-21206 RRID:AB-2535792, #A-10037 RRID:AB-2534013, #A-10042 RRID:AB-2757564). DNA was stained using Hoechst 33342 (0.002% wt:vol, Sigma, Saint Louis, Missouri, USA). Fluorescent tags were visualized using a confocal microscope (LSM 5 Live Duo, Zeiss). In control specimens without primary antibodies, neither Alexa 488 nor 568 fluorescent tags were observed. Immunocytochemistry analysis required 4 to 5 additional cochleae per age and strain. All experiments were performed in triplicate.

#### MBP immunodensity

The semi-quantitative analysis of MBP green immunofluorescence per section was obtained using a custom-made software written in Matlab (Mathworks) and expressed as arbitrary units. MBP immunodensity was assessed by measuring the mean green value, subtracted by the mean value of the background staining. The average value from at least 3 sections of the cochlear basal region of each animal was defined as the average MBP immunofluorescence for each animal and was then averaged for each group (*n* = 4 cochleae per age and strain).

#### Macrophage counts

The time course of macrophage numbers was assessed in the transverse sections of the cochlear basal regions of both WT and p43^−/−^ mice aged 1, 6, and 12 months. To assess macrophages per section, CD45-labeled macrophages were counted in five areas of each cochlea: the spiral ganglion, the spiral lamina, the organ of Corti, the spiral ligament, and the stria vascularis (see Fig. 6a). The average value in at least 4 or 5 sections from a cochlear basal region of each animal was defined as the average number of macrophages per section for each animal and then was averaged for each group (*n* = 4 cochleae per age and strain).

#### Nuclear NF-κB-positive SGN counts

The time course of nuclear NF-κB-positive SGNs was assessed in transverse sections of Rosenthal’s canal of cochlear basal regions from both WT and p43^−/−^ aged 1, 6, and 12 months. The percentage of calbindin-positive SGNs with nuclear staining of NF-κB was recorded as nuclear NF-κB-positive SGNs. The average value in 4 or 5 sections from each animal was defined as the percentage positive for each animal and then was averaged for each group (*n* = 5 cochleae per age and strain).

### Statistics

Data are expressed as the mean ± SD. Normality of the variables was assessed using the Shapiro-Wilks test. In the case of two population groups, the significance of the group difference was assessed using a Wilcoxon test. For three or more population groups, if conditions for a parametric test were met, the significance of the group differences was assessed with a one-way ANOVA; once the significance of the group differences (*P* ≤ 0.05) was established, Dunn’s tests were then used for post hoc comparisons between pairs of groups. Kruskal–Wallis tests were used to assess the significance of differences among several groups. The *P* values are indicated in the legends for each figure. Based on data from our previous reports [53] or from preliminary experiments, we calculated the sample size using G*Power 3.1.9.2 to ensure adequate power of key experiments for detecting pre-specified effect sizes.

## Supplementary Information

**Additional file 1: Figure S1.** TRα expression. **Figure S2.** Functional and morphological changes p43^-/^- mice. **Figure S3.** LC3B staining. **Figure S4.** Noise-induced threshold shift only partially recovers in P43^−/−^ mice 15 days after exposure. **Figure S5.** P43 deletion leads to enhanced ARHL.

**Additional file 2.** Individual values for figures. Each sheet in the Excel-file is named by the figure.

## Data Availability

All data generated or analyzed during this study are included in this published article and its supplementary information files. The individual data values are provided in Additional file 2.

## Abbreviations

TRα: Thyroid hormone receptor alpha
TRβ: Thyroid hormone receptor beta
ARHL: Age-related hearing loss
T3: Triiodothyronine
OHC: Outer hair cell
SGNs: Spiral ganglion neurons
DPOAE: Distortion-product otoacoustic emission
ABR: Auditory brainstem response
EP: Endocochlear potential
SEM: Scanning electron microscopy
TEM: Transmission electron microscopy
IHC: Inner hair cell
