# Supplementary material for: Exacerbated age-related hearing loss in mice lacking the p43 mitochondrial T3 receptor
Source: BMC Biol. 2021 Feb 1;19:18. doi: 10.1186/s12915-021-00953-1 (PMC7852282; doi:10.1186/s12915-021-00953-1)
Supplement: Supplementary file 1 — Additional file 1: Figure S1. TRα expression. Figure S2. Functional and morphological changes p43-/- mice. Figure S3. LC3B staining. Figure S4. Noise-induced threshold shift only partially recovers in P43−/− mice 15 days after exposure. Figure S5. P43 deletion leads to enhanced ARHL. [file 12915_2021_953_MOESM1_ESM.zip › Additional file 1-final.docx]

**Supplementary Information**

**Additional file 1 of Exacerbated age-related hearing loss in mice lacking the p43 mitochondrial T3 receptor**

1. **Additional File 1: Figure S1-S5**

**
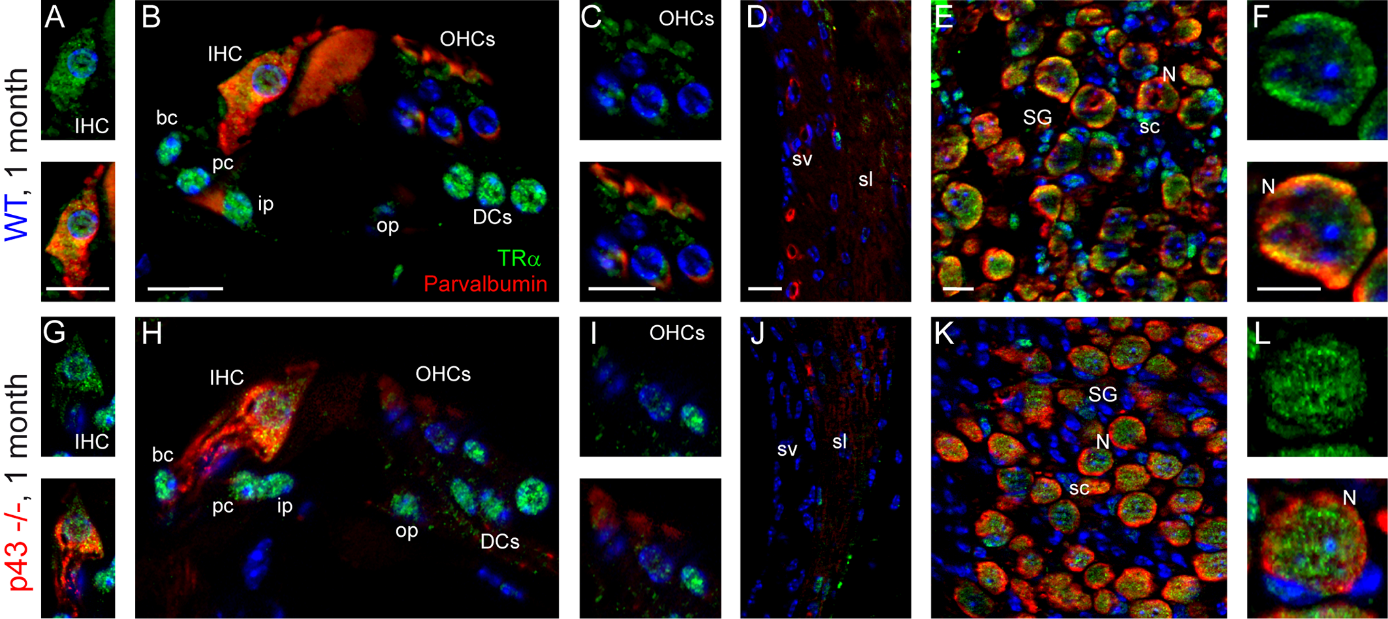
**

**Additional file 1: FigS1. TRα expression**

**A-L**: Confocal images of transverse cryostat sections of the organ of Corti (**B, H**), stria vascularis (**D**, **J**), and SGNs (**E, K**), from WT (**A**-**F**) and p43^-/-^ (**G**-**L**) mice at 1 month**.** The sections were immunolabeled for thyroid hormone receptor α1, 2 (TRα, green), Parvalbumin (red) and Hoechst (blue). **A**, **C**, **F**, **G**, **I** and **L**: Higher magnification images showing TRα immunoreactivity in IHCs (**A**, **G**), OHCs (**C**, **I**), and SGNs (**F**, **L**) from WT (**A**, **C** and **F**) and p43^-/-^ (**G**, **I** and **L**). Note that P43^-/-^ mice exhibited a reduction in TRα immunoreactivity mainly in the cytoplasm of OHCs and IHCs, and to a lesser extent in that of SGNs. bc: border cell, pc: phalangeal cell, ip: inner pillar cell, op: outer pillar cell, DCs: Deiters cells, sv: stria vascularis, sl: spiral ligament, sc: satellite cell, N: neuron. Scale bars = 10 µm.

**
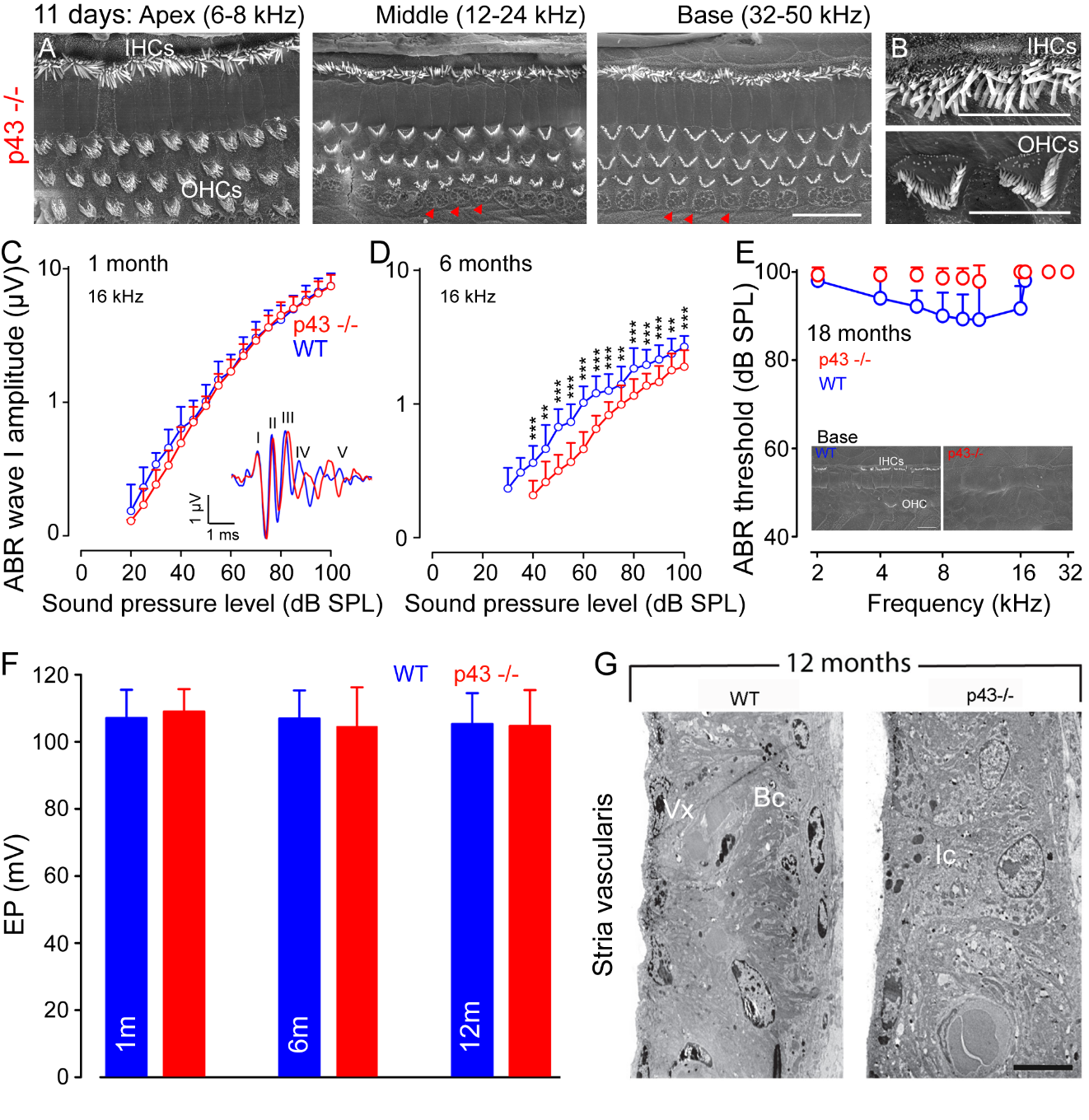
**

**Additional file 1: FigS2. Functional and morphological changes p43^-/-^ mice**

**A**-**B**: Representative scanning electron microscopy (SEM) micrographs showing the apical, middle, and basal cochlear regions from p43^-/-^  at 11 days. Note the traces of the former positions of the marginal pillars (indicated by red arrows). Scale bars: **A** = 25 µm, **B** = 10 µm. **C-D:** ABR Peak I amplitude (μV) as a function of tone-burst sound pressure level for 16 kHz for p43^-/-^ (red plot) and WT (blue plot) mice aged 1 (**C**) and 6 (**D**) months. **Inset** in **C**: mean ABR waveforms evoked by a 16 kHz tone burst with stimulus pressure at 70dB SPL. Data are means ± SD (1 month: WT: n=14, p43^-/-^ : n=8; 6 months: WT: n=26, p43^-/-^ : n=28). One-way ANOVA test was followed by Dunn’s test, ***P* ≤ 0.01, ****P* ≤ 0.001. **E:** ABR thresholds recordings in p43^-/-^ (red plot) and WT (blue plot) mice aged 18 months. Data are means ± SD (n =7 per strain). **Inset** in **E**: SEM micrographs showing the basal cochlear regions from WT and p43^-/-^  mice aged 18 months. Scale bar = 10 µm. **F**: Endocochlear potential (EP) recordings in p43^-/-^, and WT mice at different ages. Data are expressed as mean ± SD (n = 6 to 8 per age per strain). **G:** Representative transmission electron micrographs show the stria vascularis from WT (left) and p43^-/-^ (right) mice at 12 months. Note that the three layers of strial cells; marginal (Mc), intermediate (Ic), and basal (Bc) cells, and the blood vessels (Vx) appear normal. Scale bar = 10 µm.

**
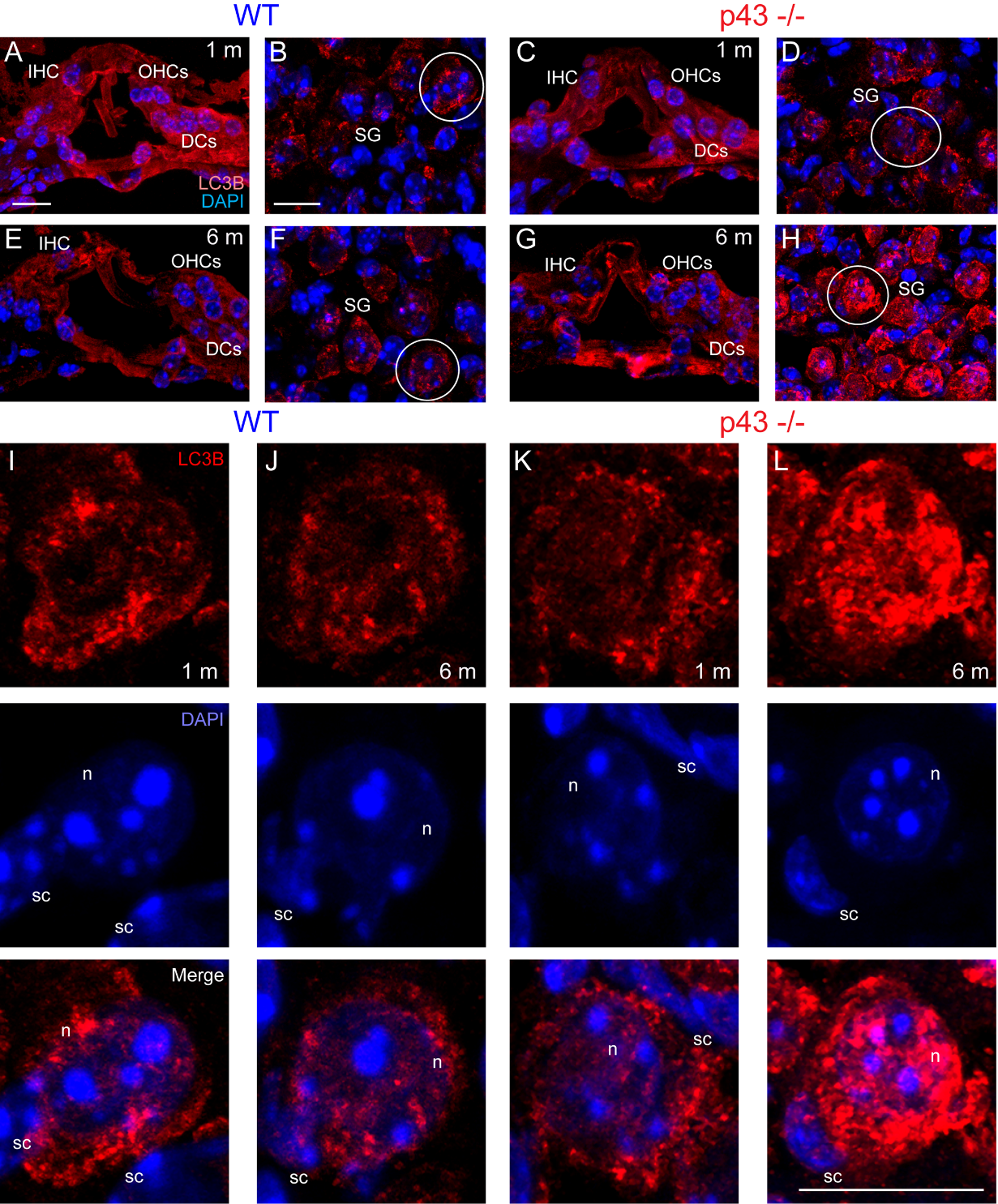
**

**Additional file 1: FigS3. LC3B staining**

**A**-**L**: confocal images of cryostat sections of the organ of Corti (**A**, **C, E** and **G**) and spiral ganglion (**B**, **D, F** and **H**). The sections were labeled for LC3B (red) and counter-stained with Hoechst (blue). **I-L**: higher magnification images showing representative SGNs derived from white circled areas in **B**, **D**, **F** and **H**. Note the diffuse pattern of LC3 staining in the organ of Corti (**A**, **C, E** and **G**) and punctate staining for LC3 in the SGNs and their Schwann cells (**B**, **D, F** and **H** and **I-L**). SGNs are identifiable by their larger, more weakly stained, spherical nuclei, whereas the nuclei of Schwann cells are recognized by their irregular shape and more intense staining (middle panels in **I-L**). DCs: Deiters cells, sc: Schwann cells, n: neuron. Scale bars: 20 µm, n = 4 to 5 cochleae per age and strain.

**
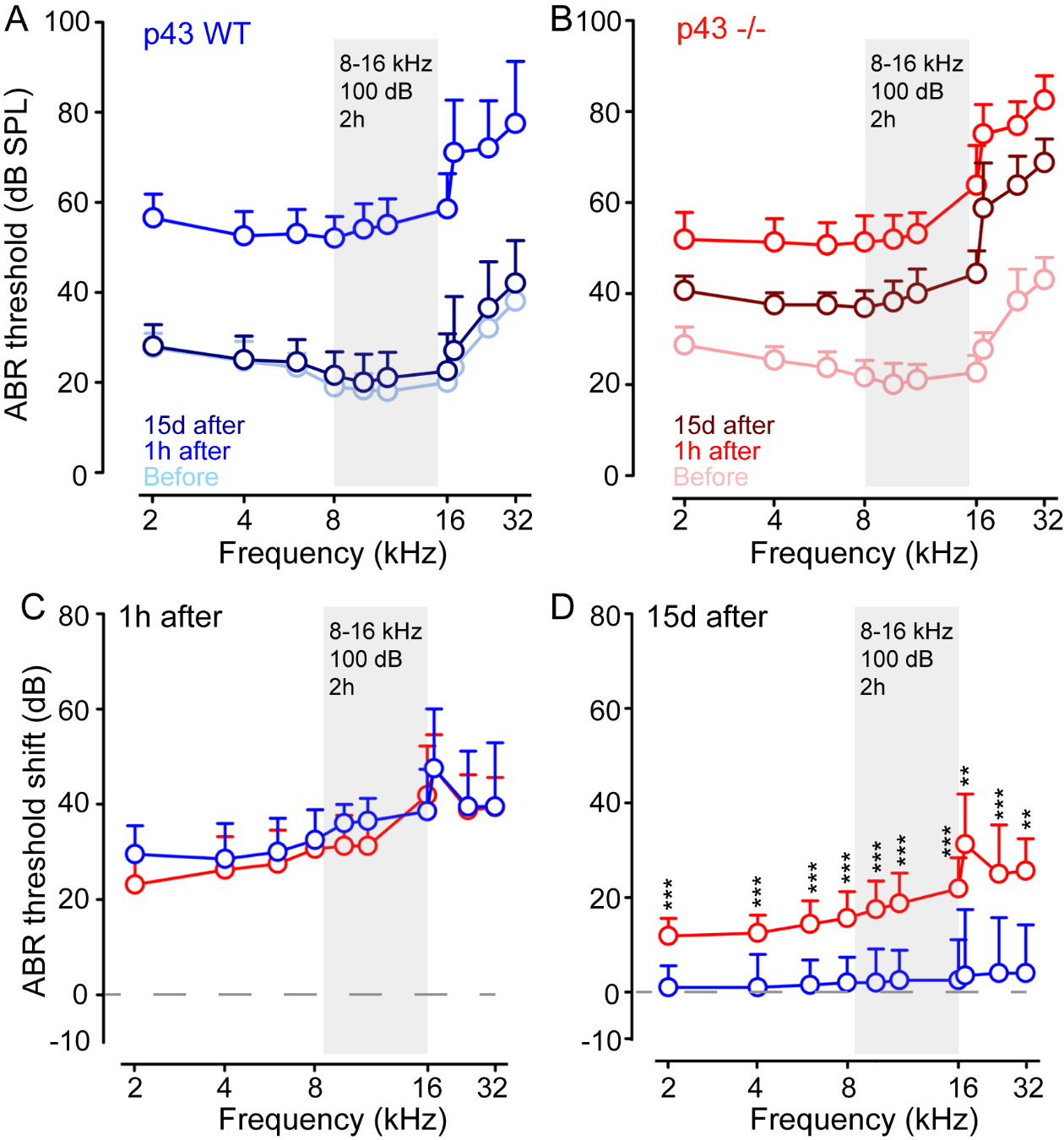
**

**Additional file 1: FigS4. Noise-induced threshold shift only partially recovers in P43^-/-^ mice 15 days after exposure**

**A-B**: ABR thresholds in WT (**A**) and p43^-/-^ (**B**), measured before, 1 hour and 15 days after exposure to an 8–16 kHz octave band noise at 100 dB for 2 hours. **C-D**: Noise-induced threshold shift in WT (blue plot) and p43^-/-^ (red plot) mice 1 hour (**C**) and 15 days (**D**) after noise exposure. Note that hearing thresholds before and the initial temporary threshold shift 1 hour after noise exposure were similar in both strains. At 15 days after exposure, an almost complete recovery of hearing threshold was seen in WT mice, whereas only a partial recovery of hearing threshold was observed in p43^-/-^ mice. Data are means ± SD (n=8 to 15 per age, strain and time after exposure). One-way ANOVA test was followed by Dunn’s test, ****P* ≤ 0.001.

**
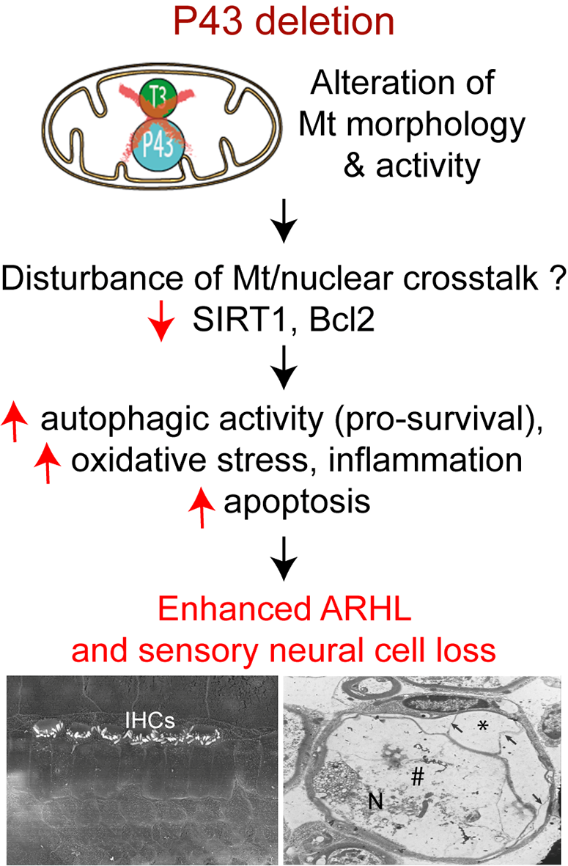
**

**Additional file 1: FigS5. P43 deletion leads to enhanced ARHL**

P43^−/−^ mice display an alteration of mitochondrial morphology and function, a drastic fall of SIRT1 and Bcl2 expression, and a subsequent increase of autophagic activity, oxidative stress, inflammation, and apoptosis, together leading to enhanced ARHL and sensory-neural cell death in the cochlea.
