# Supplementary figures and images for: Exacerbated age-related hearing loss in mice lacking the p43 mitochondrial T3 receptor
Source: BMC Biol. 2021 Feb 1;19:18. doi: 10.1186/s12915-021-00953-1 (PMC7852282; doi:10.1186/s12915-021-00953-1)

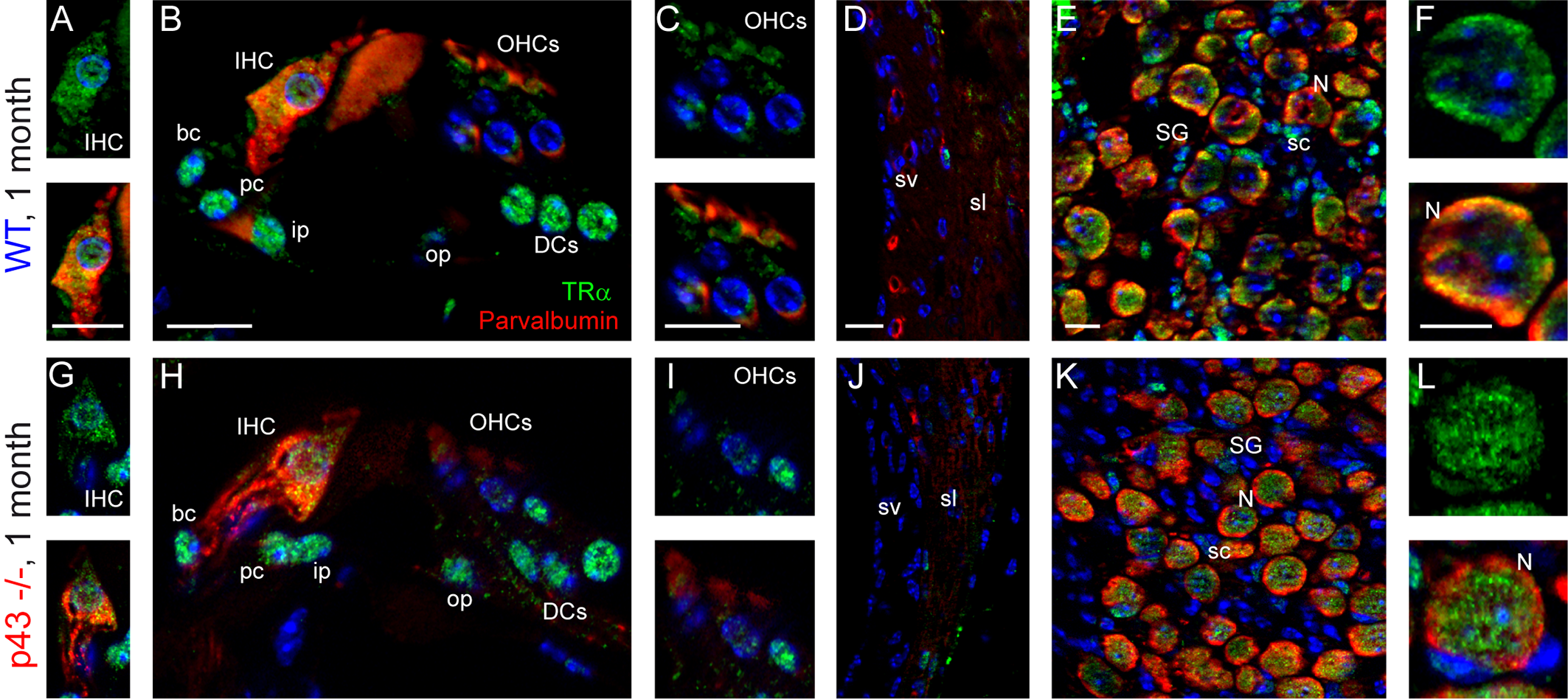

Supplement: Supplementary file 1 — Additional file 1: Figure S1. TRα expression. Figure S2. Functional and morphological changes p43-/- mice. Figure S3. LC3B staining. Figure S4. Noise-induced threshold shift only partially recovers in P43−/− mice 15 days after exposure. Figure S5. P43 deletion leads to enhanced ARHL. [file 12915_2021_953_MOESM1_ESM.zip › Additional file 1 FigS1.tif]

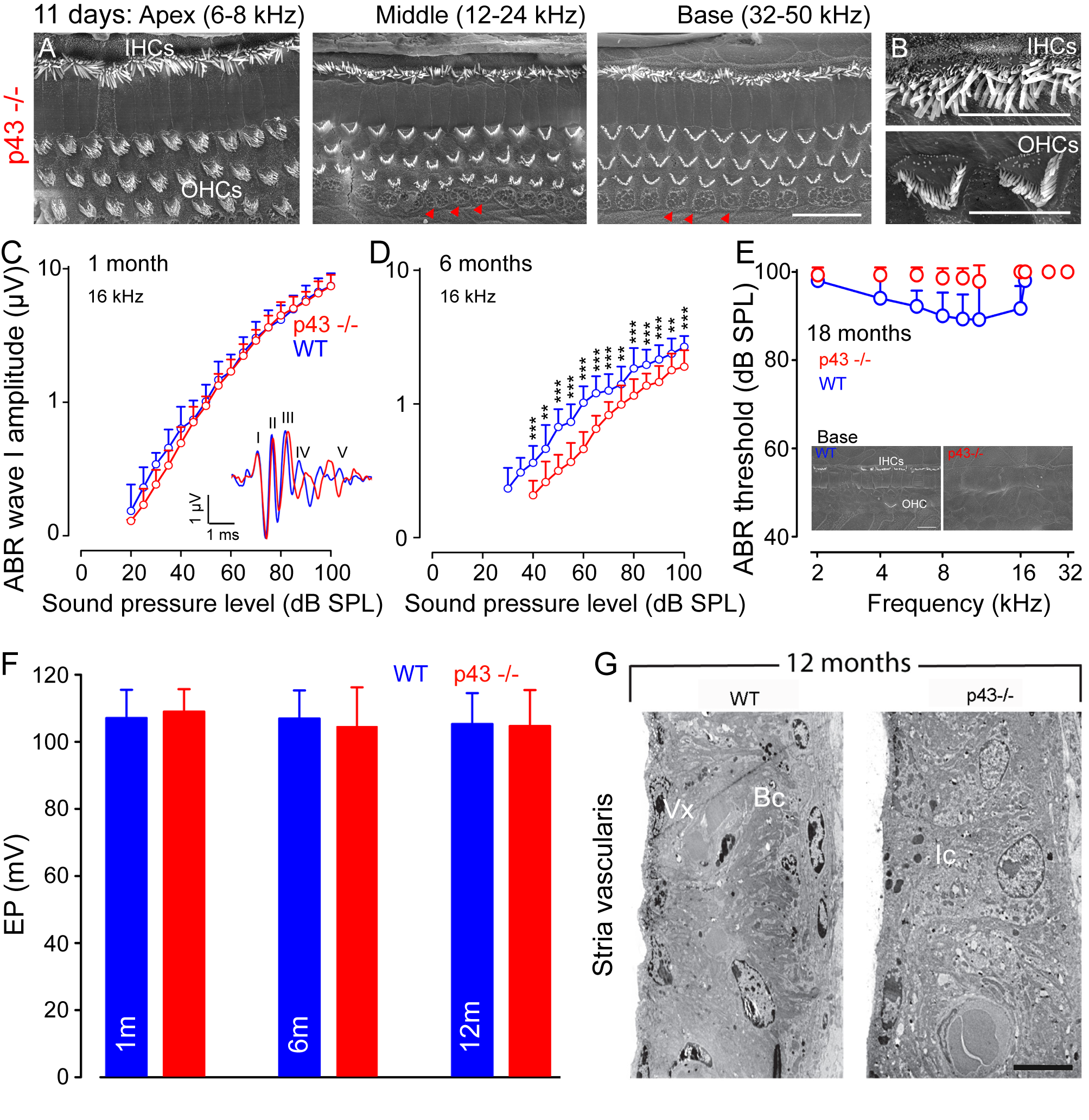

Supplement: Supplementary file 1 — Additional file 1: Figure S1. TRα expression. Figure S2. Functional and morphological changes p43-/- mice. Figure S3. LC3B staining. Figure S4. Noise-induced threshold shift only partially recovers in P43−/− mice 15 days after exposure. Figure S5. P43 deletion leads to enhanced ARHL. [file 12915_2021_953_MOESM1_ESM.zip › Additional file 1 FigS2.tif]

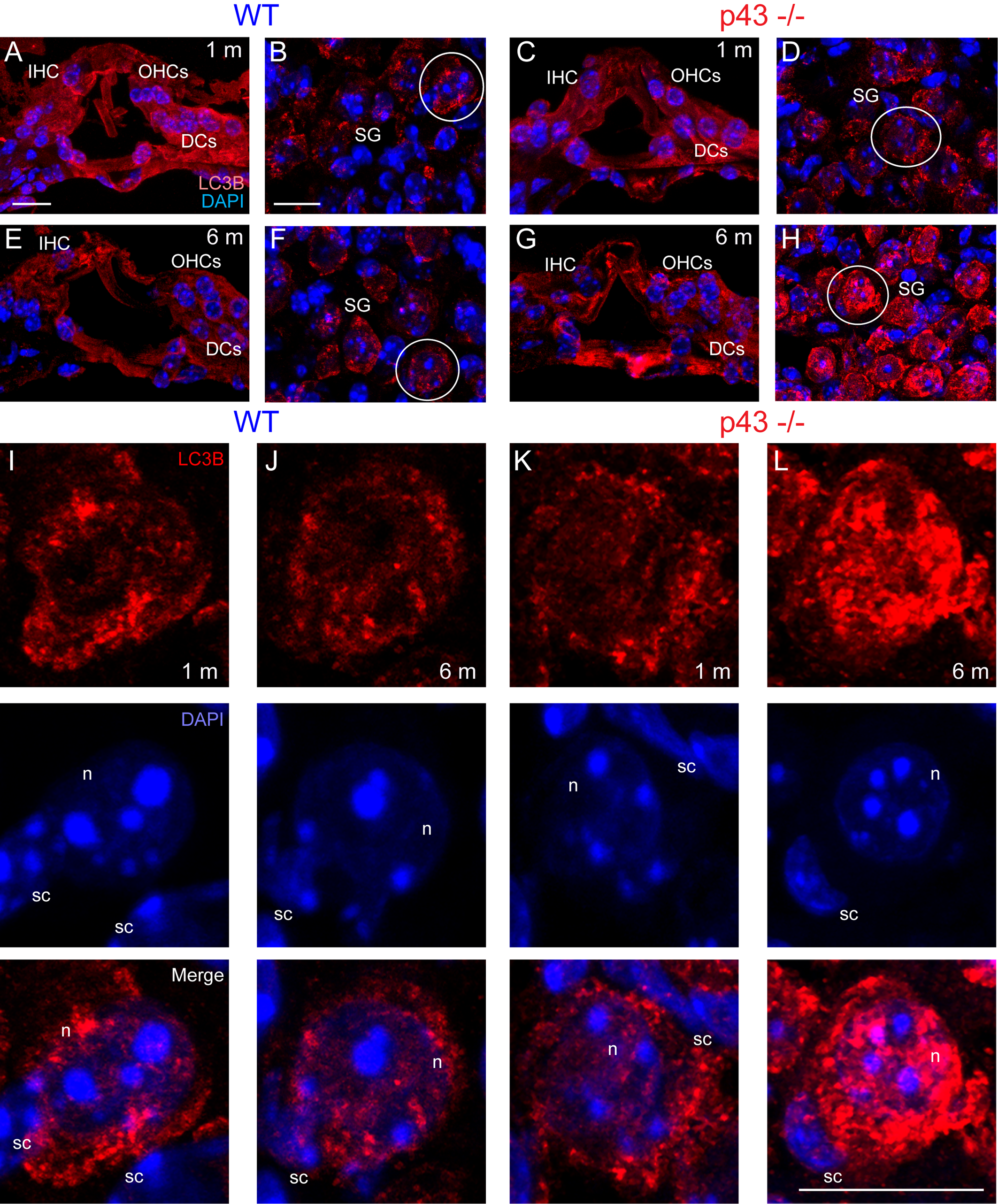

Supplement: Supplementary file 1 — Additional file 1: Figure S1. TRα expression. Figure S2. Functional and morphological changes p43-/- mice. Figure S3. LC3B staining. Figure S4. Noise-induced threshold shift only partially recovers in P43−/− mice 15 days after exposure. Figure S5. P43 deletion leads to enhanced ARHL. [file 12915_2021_953_MOESM1_ESM.zip › Additional file 1 FigS3.tif]

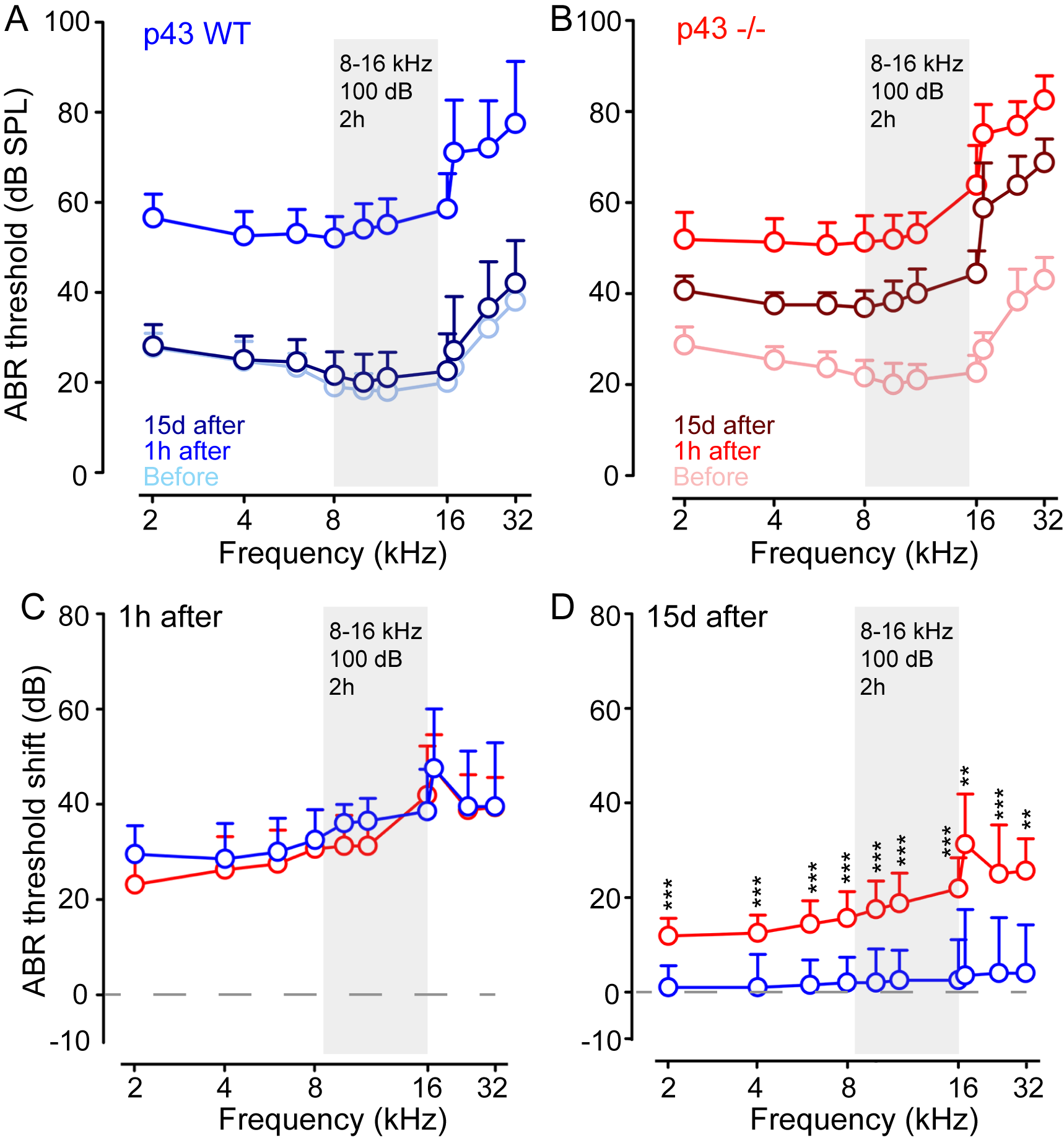

Supplement: Supplementary file 1 — Additional file 1: Figure S1. TRα expression. Figure S2. Functional and morphological changes p43-/- mice. Figure S3. LC3B staining. Figure S4. Noise-induced threshold shift only partially recovers in P43−/− mice 15 days after exposure. Figure S5. P43 deletion leads to enhanced ARHL. [file 12915_2021_953_MOESM1_ESM.zip › Additional file 1 FigS4.tif]

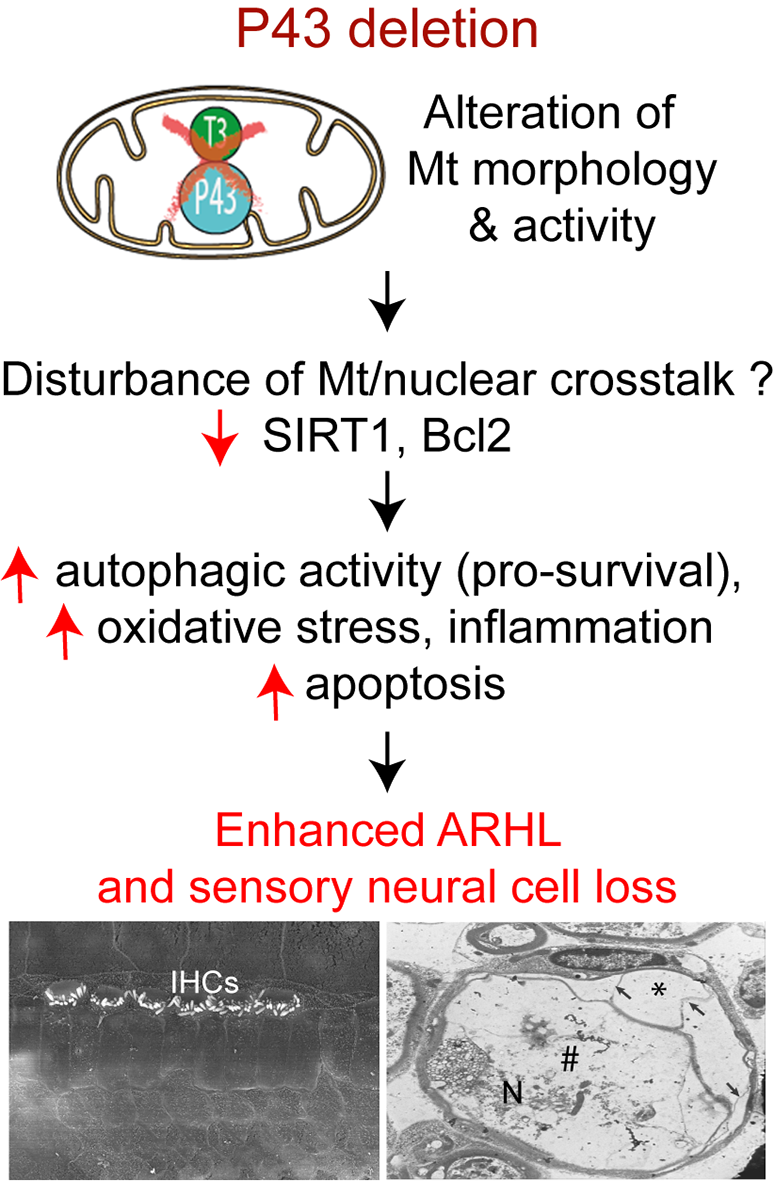

Supplement: Supplementary file 1 — Additional file 1: Figure S1. TRα expression. Figure S2. Functional and morphological changes p43-/- mice. Figure S3. LC3B staining. Figure S4. Noise-induced threshold shift only partially recovers in P43−/− mice 15 days after exposure. Figure S5. P43 deletion leads to enhanced ARHL. [file 12915_2021_953_MOESM1_ESM.zip › Additional file 1 FigS5.tif]
